# Masks for COVID‐19

**DOI:** 10.1002/advs.202102189

**Published:** 2021-11-26

**Authors:** Wei Deng, Yajun Sun, Xiaoxue Yao, Karpagam Subramanian, Chen Ling, Hongbo Wang, Shauhrat S. Chopra, Ben Bin Xu, Jie‐Xin Wang, Jian‐Feng Chen, Dan Wang, Honeyfer Amancio, Stevin Pramana, Ruquan Ye, Steven Wang

**Affiliations:** ^1^ Department of Mechanical Engineering City University of Hong Kong Hong Kong 999077 China; ^2^ School of Energy and Environment City University of Hong Kong Hong Kong 999077 China; ^3^ Department of Mechanical and Construction Engineering Northumbria University Newcastle upon Tyne NE1 8ST UK; ^4^ State Key Laboratory of Organic Inorganic Composites Beijing University of Chemical Technology Beijing 100029 China; ^5^ Department of Chemical Engineering and Biotechnology Cambridge University Cambridge CB2 1TN UK; ^6^ School of Engineering Newcastle University Newcastle upon Tyne NE1 7RU UK; ^7^ Department of Chemistry City University of Hong Kong Hong Kong 999077 China

**Keywords:** antimicrobial materials, COVID‐19, graphene, photothermal, SARS‐CoV‐2, substitutes, superhydrophobic, triboelectric nanogenerators

## Abstract

Sustainable solutions on fabricating and using a face mask to block the severe acute respiratory syndrome coronavirus 2 (SARS‐CoV‐2) spread during this coronavirus pandemic of 2019 (COVID‐19) are required as society is directed by the World Health Organization (WHO) toward wearing it, resulting in an increasingly huge demand with over 4 000 000 000 masks used per day globally. Herein, various new mask technologies and advanced materials are reviewed to deal with critical shortages, cross‐infection, and secondary transmission risk of masks. A number of countries have used cloth masks and 3D‐printed masks as substitutes, whose filtration efficiencies can be improved by using nanofibers or mixing other polymers into them. Since 2020, researchers continue to improve the performance of masks by adding various functionalities, for example using metal nanoparticles and herbal extracts to inactivate pathogens, using graphene to make masks photothermal and superhydrophobic, and using triboelectric nanogenerator (TENG) to prolong mask lifetime. The recent advances in material technology have led to the development of antimicrobial coatings, which are introduced in this review. When incorporated into masks, these advanced materials and technologies can aid in the prevention of secondary transmission of the virus.

## Graphical abstract

Masks are critical during the coronavirus pandemic of 2019 (COVID‐19), and there is a huge demand for them globally. Many countries use cloth masks and 3D‐printed masks as substitutes, but the filtration efficiencies are unqualified. Herein, various new mask technologies and advanced materials, including metal nanoparticles, graphene, metal organic framework (MOF), and triboelectric nanogenerator (TENG) are reviewed, to deal with critical shortages, cross‐infection, and secondary transmission risk of masks.

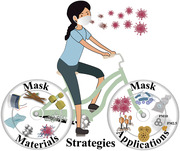

## 1 Introduction

### 1.1 Why Masks are Important during COVID‐19 Pandemic

The coronavirus pandemic of 2019, abbreviated as COVID‐19,^[^ ^1^ ^]^ has been considered as an unprecedented healthcare crisis since the Spanish Flu pandemic in early 20th century, which has severely disrupted nearly every aspect of daily life. As of 21th March 2021, the World Health Organization (WHO) has reported over 122 million confirmed cases of COVID‐19, with a death toll of over 2.7 million.^[^ ^2^ ^]^ The primary cause of this disease is the severe acute respiratory syndrome coronavirus 2 (SARS‐CoV‐2), which causes infected individuals to manifest flu‐like symptoms.^[^ ^3^, ^4^ ^]^ These symptoms include dry coughs, chest pains, fevers, anosmia, and in the most severe cases, pneumonia, and acute respiratory distress syndrome (ARDS) and even death.^[^ ^5^, ^6^, ^7^, ^8^, ^9^ ^]^ It has been shown that SARS‐CoV‐2 is highly infectious and has an incubation period typically lasting from 1 to 14 days with some special cases exceeding 14 days.^[^ ^10^, ^11^, ^12^ ^]^ Furthermore, studies have shown the existence of asymptomatic carriers of the virus which leads to issues to contain the virus transmission.^[^ ^13^, ^14^, ^15^ ^]^ All these characteristics of COVID‐19 have dramatically made it more difficult to detect, monitor, and prevent its spread.

**Figure 1:**
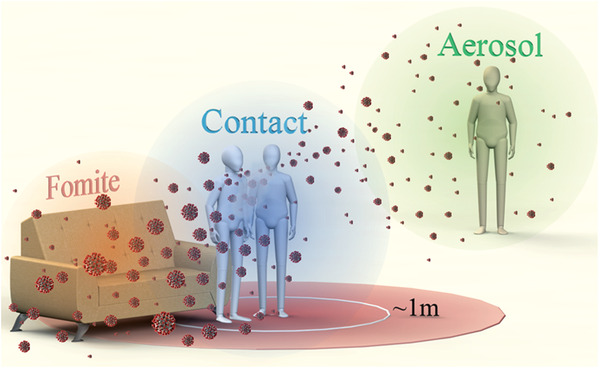
Schematic of three different pathways for the transmission of respiratory diseases. Contact transmission is a result of direct physical contact with an infected person, e.g., handshaking, and the virus is transferred to them.^[^ ^16^, ^25^ ^]^ Fomite transmission is an indirect and subtle pathway whereby large droplets settle on surface, such as door handles, tabletops, and buttons etc., which then becomes a fomite resource.^[^ ^25^, ^26^, ^27^ ^]^ Aerosol transmission can result in the wide spread of virus with the air flow.

**Figure 2:**
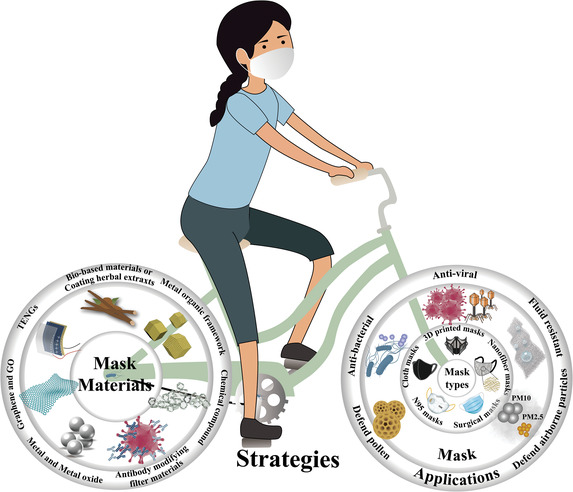
Graphical expression of materials used in masks and their applications. There are different types of masks that can be used which rely on the basic filtration effect to trap aerosol particles during the COVID‐19 pandemic as shown by the inner circle of right wheel. Through modifying the masks with various functional materials or technologies as shown by the left wheel, it makes masks possess various functions such as antiviral and hydrophobic abilities shown in the outermost circle of the right wheel, which are significantly useful to help the world fight COVID‐19.

The general transmission pathway for COVID‐19, like other respiratory diseases, including influenza, Severe Acute Respiratory Syndrome (SARS), and Middle East Respiratory Syndrome (MERS), consists of contact transmission, fomites transmission and aerosol transmission,^[^ ^16^, ^17^, ^18^, ^19^, ^20^ ^]^ as shown in **Figure** **1**. All these modes of transmission are thought to involve SARS‐CoV‐2‐laden respiratory fluid droplets which are expelled by the infected person whenever they perform respiratory‐related activities such as when they cough, sneeze, speak, sing, or even breathe.^[^ ^21^, ^22^, ^23^, ^24^, ^25^ ^]^ Viruses in these droplets can remain viable and infectious for extended periods of time and then result in the spread of the infection.^[^ ^26^, ^27^, ^28^, ^29^ ^]^ Respiratory fluid droplets typically include coarse particles (>5 µm) and fine particles (<5 µm).^[^ ^5^, ^17^, ^18^ ^]^ Coarse particles are reported to have a relatively short transport range, ≈1 m, and settle quickly due to gravitational effects, which can then lead to contact and fomites transmission.^[^ ^30^, ^31^, ^32^ ^]^ However, fine particles containing viruses can potentially become suspended aerosol particles and stay in air for prolonged period of time, allowing the virus to be transmitted via aerosols over long distances (> 1 m),^[^ ^20^, ^33^, ^34^ ^]^ i.e., aerosol transmission. Therefore, aside from measures such as consistently practicing good hygiene and establishing social distancing to avoid former two pathways, preventing the spread of the virus through aerosols is also very crucial through other infection prevention and control measures such as wearing mask, quarantine, and isolation.^[^ ^24^, ^35^ ^]^ Hereinto, face masks or respirators can be an effective and essential equipment to protect healthcare workers and members of the general public who may be exposed to the virus.^[^ ^18^, ^33^, ^36^, ^37^, ^38^, ^39^, ^40^ ^]^ From epidemiological data, places, where the spread of COVID‐19 has been most effectively controlled, have implemented the measure of universal mask wearing, such as China, Singapore, and South Korea.^[^ ^18^, ^41^, ^42^, ^43^, ^44^, ^45^, ^46^ ^]^ Hence, face masks and respirators, especially these with some special functions such as antiviral ability, superhydrophobicity, reusable and recharging capacities as shown in **Figure** **2**, play a vital role to effectively control the spread of COVID‐19.

### 1.2 Face Masks and Respirators

**Figure 3:**
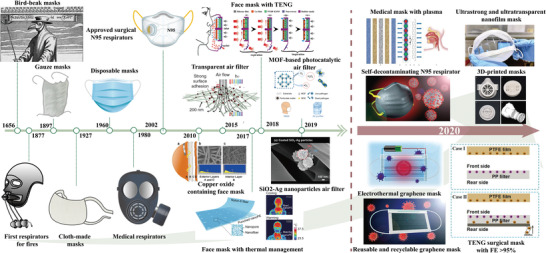
The timeline in the development of mask technologies from its introduction in the 17th century to modern‐day and future multifunctional applications. Copper oxide containing face mask: Reproduced under the terms of the Creative Commons Attribution License.^[^ ^190^ ^]^ Copyright 2010, Borkow. Transparent air filter: Reproduced with permission.^[^ ^226^ ^]^ Copyright 2015, Springer Nature. Face mask with the thermal management: Adapted with permission.^[^ ^303^ ^]^ Copyright 2017, American Chemical Society. Face mask with TENG: Adapted with permission.^[^ ^290^ ^]^ Copyright 2018 American Chemical Society. SiO_2_‐Ag nanoparticles air filter: Adapted under the terms of the Creative Commons Attribution 4.0 International License.^[^ ^199^ ^]^ Copyright 2019, Taiwan Association for Aerosol Research. MOF‐based photocatalytic air filter: Adapted under the terms of the Creative Commons Attribution 4.0 International License.^[^ ^247^ ^]^ Copyright 2019, The Authors. Published by Springer Nature. Medical masks with plasma: Adapted under the terms of the Creative Commons Attribution 4.0 International License.^[^ ^321^ ^]^ Ultrastrong and ultratransparent nanofilm mask: Adapted under the terms of the Creative Commons Attribution 4.0 International License.^[^ ^305^ ^]^ Self‐decontaminating N95 respirator: Reproduced with permission.^[^ ^216^ ^]^ Copyright 2020, American Chemical Society. 3D‐printed masks: Reproduced with permission.^[^ ^140^ ^]^ Copyright 2020, Elsevier. Electrothermal graphene mask: Adapted with permission.^[^ ^225^ ^]^ Copyright 2020, American Chemical Society. TENG surgical mask with FE >95%: Reproduced with permission.^[^ ^291^ ^]^ Copyright 2021, Elsevier. Reusable and recyclable graphene mask: Adapted with permission.^[^ ^215^ ^]^ Copyright 2020, American Chemical Society.

**Figure 4:**
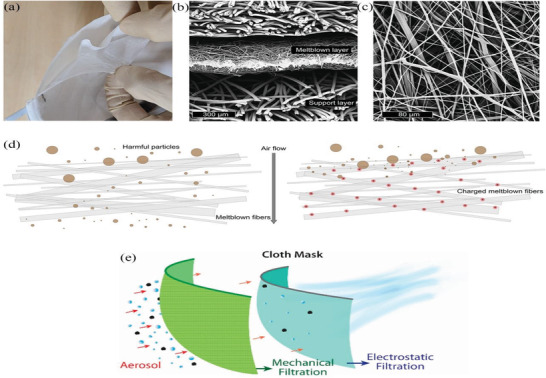
Structure diagram of N95 respirators. a) Peeling apart a representative N95 respirator reveals multiple layers of nonwoven materials. b) Scanning electron microscope (SEM) cross‐section image reveals that the middle meltblown layer has finer fibers with thickness around 300 µm. c) SEM image of meltblown fibers reveals a complicated randomly oriented network of fibers with diameters ≈1–10 µm. d) Schematic illustration of meltblown fibers (left) without and (right) with electret charging. In the left figure, smaller particles are able to pass through to the user, but they are electrostatically captured in the case of an electret (right). a‐d) Reproduced with permission.^[^ ^5^ ^]^ Copyright 2020, American Chemical Society. e) Schematic illustration of homemade cloth masks obtained by combining different fabric materials. Reproduced with permission.^[^ ^90^ ^]^ Copyright 2020, American Chemical Society.

Since the first time use of mask in history, masks have undergone constant development and improvement, as shown in **Figure** **3**. At present, the most commonly used face masks and respirators are, respectively, surgical masks and N95 level respirators, which are fabricated from synthetic or natural polymers or composites, typically polypropylene (PP), polyethylene (PE), glass papers, and woolen felt.^[^ ^47^, ^48^, ^49^ ^]^ From these materials, melt‐blown nonwovens, especially nonwoven PP fabric, are the most commonly used.^[^ ^50^, ^51^, ^52^ ^]^ In general, face masks and respirators consist of three layers: a middle filter layer – the most important layer with regards to protection from particles or droplets carrying viruses and bacteria, and two external layers, as shown as **Figure** **4**a. The filter layer and external layers are fabricated using nonwoven meltblown polypropylene and spun bond polypropylene, respectively, which leads to the former having smaller and denser fibers and hence smaller voids compared to the latter. Some N95 level respirators have additional layers to provide better leak protection and filtration performance – for example, a support layer to provide shape rigidity for the respirator.

In typical surgical masks and respirators, the meltblown filter layer, comprising PP microfibers of diameter ≈1‐10 µm, has a thickness of 100–1000 µm, as shown in Figure 4b,c.^[^ ^5^ ^]^ This, however, still leaves many gaps and voids in the microfiber‐network, leading to high air permeability and a porosity of up to 90%.^[^ ^53^ ^]^ This structure of the filter layer results in an inadequate filtration efficiency of fine aerosol particles, and only large aerosol particles can be blocked. Therefore, to address this issue of improving filtration performance without sacrificing good air permeability, the filter layer is charged through some charging technologies such as corona discharge, induction charging, and triboelectric techniques during fabrication into quasi‐permanent dipoles called electrets, in which corona discharge technique is the most commonly used one.^[^ ^52^, ^54^, ^55^, ^56^, ^57^, ^58^, ^59^ ^]^ These electrets can absorb fine aerosols, which can significantly increase the filtration efficiency through the mechanism of electrostatic interaction,^[^ ^60^, ^61^, ^62^, ^63^, ^64^ ^]^ as shown in Figure 4d.

Face mask and respirator structure are designed so that aerosol particles can be filtered out using a combination of the following five mechanisms – 1) gravity settling, 2) inertial impaction on the fibers, 3) interception by the fibers, 4) diffusion, and 5) electrostatic attraction.^[^ ^65^, ^66^, ^67^, ^68^ ^]^ For aerosol particles >1 µm, the first two mechanisms play an important role. However, as the aerosol's particle size decreases, other mechanisms dominate the filtration process. Diffusion and mechanical interception of particles are important for particle sizes from 100 nm to 1 µm, while electrostatic attraction predominates when particle sizes are <100 nm.^[^ ^62^, ^69^, ^70^, ^71^ ^]^

Because the filtration process employs several different mechanisms, the filtration performance of the filter layer depends on many parameters including fiber conditions (fiber organization, pore size, the charge of fiber, fiber thickness and diameter, etc.) and environmental conditions (temperature, relative humidity, etc.).^[^ ^62^, ^72^ ^]^ Of these, the charge intensity of the filter layer is one of the most important parameters that affect the filtration efficiency of the mask or respirator.^[^ ^73^, ^74^ ^]^ Both the charge intensity and the strength of electrostatic attraction are dependent on the fiber material's dielectric property. In general, polymeric materials such as PE and PP make excellent choices for the fibers of masks and respirators as a result of the good properties of high electrical resistance and thermal stability.^[^ ^73^ ^]^ Furthermore, if the charge on the filter layer is reduced, the filtration performance will decrease dramatically. The diameter of the fiber also affects filtration efficiency, and fibers with small diameters result in large specific surface areas, which in turn means the voids within the fiber matrix are smaller. This increases filtration performance. However, the air permeability gets compromised.

Relative humidity is also a significant factor for the filtration efficiency: the filtration efficiency is significantly reduced when relative humidity increases.^[^ ^75^, ^76^, ^77^ ^]^ This degradation of filtration efficiency is believed to be caused by the reduction of the charge of the filter layer fibers due to direct contact with water molecules.^[^ ^5^, ^75^, ^76^ ^]^ Thus, there is a possibility that other sources of moisture, such as the wearer's breathing and sweat, may also lead to the degradation of the mask or respirator's filtration performance, and hence it may be better for a wearer not to wear a filtering facepiece respirator (FFR) or mask for too long.^[^ ^72^, ^78^ ^]^

Face masks and respirators are required to meet certain standards before they can be used. A popular standard is given by the American Society of Testing and Materials (ASTM), whose F2100 standard sets four defined performance characteristics that potential face mask materials must comply with in order to be used.^[^ ^79^ ^]^ These are, namely, the filtration efficiency (FE), the differential pressure (∆*P*, representing air permeability), fluid resistance, and flammability. Filtration efficiency includes particulate filtration efficiency (PFE), bacterial filtration efficiency (BFE), and virus filtration efficiency (VFE). Based on the performance of masks using these four characteristics, face masks may be grouped according to different levels. For example, for the lowest standard for one common surgical mask, it is necessary to keep a minimum FE of 95%,^[^ ^80^, ^81^, ^82^, ^83^, ^84^, ^85^ ^]^ fluid resistance of 80 mmHg,^[^ ^86^, ^87^ ^]^ a maximum differential pressure of 5.0 mmH_2_O cm^−2[^ ^87^, ^88^ ^]^ or 49 Pa cm^−2^,^[^ ^35^ ^]^ and Class 1 flammability^[^ ^79^, ^89^ ^]^ in their test conditions. In addition to the four criteria, fit is also a significant factor that determines how well the face mask or respirator protects its wearer.^[^ ^90^, ^91^, ^92^ ^]^ In general, face masks perform poorly in both filtration efficiency and fit, but has better air permeability in comparison to respirators.^[^ ^93^, ^94^ ^]^

## 2 Choices of Common Face Mask and Respirator

However, the current global COVID‐19 pandemic has caused an enormous surge in the demand for surgical face masks and respirators, especially among healthcare workers, and this has caused the critical shortage of both products and raw materials. Therefore, it is urgent that some measures are taken to improve the current situation. One strategy to alleviate the critical problem involves finding substitutes for face masks and respirators, for instance, fabricating masks with other materials or using homemade face masks.

### 2.1 Cloth Masks

To combat the spread of COVID‐19 and the subsequent shortage of commercial masks, wearing cloth masks is advised for the general public in many countries and areas, especially in low‐income countries.^[^ ^85^ ^]^ Due to the abundance and cheapness of their raw materials, cloth masks have become popular, many of which are homemade using common cloth products. Cloth masks may differ from medical‐grade masks that are commercially available with regards to their material of construction and efficacy, but based on how SARS‐CoV‐2 spreads, it is still better to wear a simple cloth mask that provides some physical barrier than none at all.^[^ ^91^, ^95^, ^96^, ^97^, ^98^, ^99^, ^100^ ^]^

There are varieties of available household cloth materials, including tissue paper, pillowcase, cotton fabrics, silk, chiffon, tea cloths, kitchen towels, and so on, that can be used to fabricate simple cloth masks, some of which can easily generate electrostatic interactions.^[^ ^90^, ^101^, ^102^ ^]^ The common cloth masks, including factory‐made (namely, commercial) cloth masks and homemade cloth masks, may be made of different combinations and layers of these cloth materials, layering sequences and various shapes. For the factory‐made cloth masks, the manufacturers must use the certified process by a quality management system (e.g., the International Organization for Standardization 9001, ISO 9001) and adhere to some guidance from standards organizations (e.g., the American Association of Textile Chemists and Colorists, AATCC).^[^ ^35^ ^]^ As for homemade cloth masks, they are usually made of the common and accessible household cloth materials in daily life through some simple cutting and stitching to combine several cloth layers with stretchable ear loops by people themselves.^[^ ^103^, ^104^, ^105^ ^]^ The fabrication process is not complicated; however, few design rules must be followed: highly porous materials including gauze and elastic materials that have low filtration performance cannot be used; exhalation valves are discouraged; nose wire can be used to improve the fitness.^[^ ^35^ ^]^

As the use of cloth masks becomes more widespread, it is quite necessary to evaluate their filtration performance and the extent to which they block droplets and aerosols that contain viruses in the air, so that it can provide normative guidance for their use. Recently, researchers have performed tests on several cloth materials in terms of filtration efficiency, pressure difference, and fitness.^[^ ^106^, ^107^, ^108^, ^109^ ^]^

Konda et al. conducted a series of systematic trials and showed the filtration efficiencies of different materials that can be used to make homemade masks, including cotton, silk, chiffon, flannel, etc. These efficiencies were tested for particles that have sizes of ≈10 nm to ≈6 µm.^[^ ^90^ ^]^ NaCl‐based aerosols were used for testing the efficiency, which were generated by aerosolized NaCl solution and have been performed in other studies as one of the common test agents following ISO 16900–1 standard.^[^ ^110^ ^]^ (Other test agents include corn oil aerosols and sulfur hexafluoride gas (SF_6_) etc. Some studies also test the filtration efficiencies against nonoily and oily aerosols respectively using NaCl aerosols and dioctyl phthalate (DOP) particles.^[^ ^111^ ^]^) The filtration efficiencies achieved by the single‐layer fabrics varied, with a range of 5–95% for relatively larger particles (>300 nm), and a range of 5–80% for smaller particles (<300 nm). Among them, the single layer of cotton, cotton quilt, natural silk, and chiffon, which possess a higher thread count per inch (i.e., tighter weaves), can provide over 50% filtration efficiency, with cotton and cotton quilt materials providing over 90% filtration efficiency for particle sizes > 300 nm. However, when specific different fabrics and multiple layers were combined, they showed that there were varying degrees of improvement in filtration efficiencies, consistent with the research results of Zhao et al.^[^ ^106^ ^]^ For the four hybrid combinations of high threads‐per‐inch cotton with other materials, such as silk, chiffon or flannel, the filtration efficiency increased to >80% and >90% for particles of <300 nm and >300 nm, respectively. This drastic increase is caused by the synergistic effects of combining the mechanical filtering mechanism of cotton with the electrostatic filtering mechanism of other materials. These mechanisms are discussed in Section 1.2, and Figure 4e shows a schematic of cloth mask. Fit also plays an important role in filtration performance, with leakages leading to significant reductions (≈60%) in effectiveness.^[^ ^35^, ^90^, ^112^ ^]^ However, high thread count means less pores and smaller voids in the material and multiple layers have higher block for air, both which can lead to a high pressure difference, resulting in poor breathability. This has been proved by Zhao et al.^[^ ^106^ ^]^ and Zangmeister et al.^[^ ^107^ ^]^ All of these studies suggest that the filtration performance of cloth masks is affected by many parameters including the material features, the layers, the shapes of mask and fit, thread count, hybrid combinations, and electrostatic property of materials.

Hence, when designing cloth masks, the resulting pressure difference across the mask when in use should be minimized below 60 Pa cm^−2^, namely good breathability, and fit and leakage should also be considered to better cover the nose and mouth, meanwhile high filtration efficiency must also be ensured to meet the criterion through multi‐layers with different materials. Even so, cloth masks are not recommended as an alternative of medical masks for healthcare workers due to their inadequate filtration performance and the high risk environment.^[^ ^113^, ^114^ ^]^ According to WHO report, as alternative of medical masks for the general public and meanwhile along with other infection prevention and control measures, cloth masks are recommended to ideally consist of at least three layers like medical masks: one inner hydrophilic layer, one middle filtration layer and one outer hydrophobic layer.^[^ ^35^ ^]^ Due to contacting with the face, the hydrophilic material of inner layer should be nonirritating against the skin such as cotton, quilting cotton and flannel.^[^ ^115^ ^]^ The middle layer should be made of some materials which can improve the filtration performance, for example, some synthetic hydrophobic non‐woven materials (e.g., spunbond PP, polyester, and polyaramid)^[^ ^115^ ^]^ and the electrostatic fabrics as discussed above. The outer hydrophobic layer is used to prevent the external contamination and water from penetrating though the layer and blocking the pores of fabrics, in which these materials such as spunbond PP, polyester, or their hybrids can be used.^[^ ^115^ ^]^ Of course, if there are some advanced materials meeting the filtration requirements, the number of layers can be varied depending on the performance. In addition, with respect to future research on cloth masks, in order to realize the precise and replicable results, it is more reasonable for researchers to use the same standard parameters or methods for evaluating the filtration performance of various materials.^[^ ^116^ ^]^

Due to the critical shortage of masks, WHO also suggests to wash cloth masks in soap or detergent and then preferably soak them into hot water of at least 60 °C for 30 min.^[^ ^35^, ^117^, ^118^, ^119^ ^]^ The operating conditions including water temperature and time are believed to allow for inactivation of the viruses including SARS‐CoV‐2 on the masks.^[^ ^5^, ^120^, ^121^ ^]^ But it is better not to wash cloth mask too many times (<5×), because washing mask may result in the increase in pore size and thus decrease in filtration efficiency.^[^ ^103^ ^]^

### 2.2 3D‐Printed Masks

In addition to the use of cloth masks, researchers, and manufacturers have also paid attention to fabricating personal protective equipment (PPE), including masks and their components, by utilizing 3D printing (namely, Additive Manufacturing (AM)) to alleviate the shortage of surgical masks and respirators.^[^ ^122^, ^123^, ^124^, ^125^, ^126^, ^127^, ^128^, ^129^ ^]^ 3D printing is a novel and innovative rapid prototyping technology, and it can be used to fabricate complex geometric structures which connot be easily fabricated through traditional manufacturing process.^[^ ^130^, ^131^ ^]^ There are a host of various materials that can be used as the base material in 3D printing, such as polyamide (PA) composite, acrylonitrile butadiene styrene (ABS), nylon PA, polylactic acid (PLA), ULTEM (polyetherimide), and thermoplastic polyurethane (TPU).^[^ ^131^, ^132^, ^133^, ^134^, ^135^, ^136^, ^137^, ^138^, ^139^ ^]^

**Figure 5:**
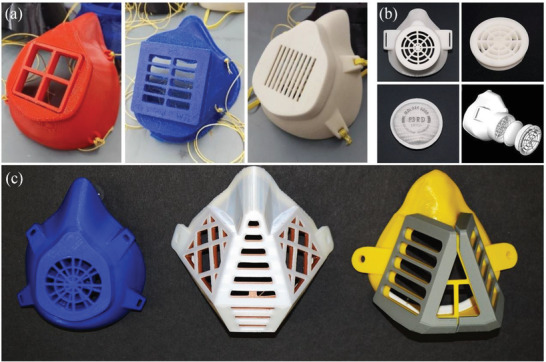
Some examples of 3D‐printed masks. a) 3D‐printed masks made using open‐sourced designs. Reproduced with permission.^[^ ^132^ ^]^ Copyright 2021, Elsevier. b) A custom‐made design for a 3D‐printed mask from. Reproduced with permission.^[^ ^140^ ^]^ Copyright 2020, Elsevier. c) The 3D‐printed masks made from PLA using fused deposition modeling (FDM) technology. Reproduced under the terms of the Creative Commons Attribution 4.0 International License.^[^ ^133^ ^]^ Copyright 2020, Vaňková et al.

Usually, a single 3D‐printed mask comprises the main reusable composite framework and the disposable filter materials, which are secured inside the framework to enable the mask to possess filtering capabilities.^[^ ^140^, ^141^, ^142^ ^]^ The time required to create a 3D‐printed mask, however, is much longer compared to commercially available masks and respirators and may take up to several hours depending on the structure, volume and the number of needed parts to fabricate the 3D‐printed mask. However, unlike common disposable face masks and respirators, 3D‐printed masks are more sustainable as they can be easily recycled and reused, which prolongs its lifetime and addresses the environmental concerns posed by medical waste generated from single‐use masks.^[^ ^143^ ^]^ For 3D‐printed masks, only the filter material requires continual replacement, which can be acquired from nonwoven meltblown products of commercial masks or commercial filter membranes.^[^ ^144^ ^]^ Based on this, there have been a variety of different types of 3D‐printed masks fabricated by researchers and manufacturers,^[^ ^132^, ^133^, ^134^, ^140^, ^141^, ^142^, ^143^, ^144^ ^]^ as shown in **Figure** **5**, and some model designs are freely available online.^[^ ^145^, ^146^, ^147^ ^]^ In the study by Bezek. et al.,^[^ ^132^ ^]^ the results showed that most of 3D‐printed masks had poor filtration performance, with only one mask possessing a >90% filtration efficiency, which is still lower than the required >95% filtration efficiency for surgical masks and N95 level respirators. These low filtration efficiencies are caused by leakages between the different interfaces on the 3D‐printed mask. Though further optimization of all the printing parameters can improve the filtration efficiency further, 3D printing process is not yet reliable or reproducible enough due to the high variability of printing conditions, processes and materials.

Although certain 3D‐printed masks may provide some protection, like cloth masks, for specific users, 3D‐printed masks may not meet the stringent standards of commercial face masks and respirators, and their filtration performance has not yet been approved by any regulatory agencies. Even so, because 3D printing is a low‐cost and rapid manufacturing technology, it is still regarded as a technology with great potential in an emerging application. However, it is not advisable that 3D‐printed masks should be used as an alternative to medical‐grade masks and respirators until enough rigorous and reliable testing for the filtration performances are conducted. All these need to be investigated further by researchers and manufacturers, especially in the areas of fit and leakage.

## 3 Improve the Filtration Efficiency

With the widespread use of masks, more and more materials are used to fabricate different types of masks, such as fabrics in cloth masks and these materials in 3D printing. However, some adopted materials may not have qualified filtration performance even though using common electret charging, because commercial masks must meet certain standards, including good filtration performance. Therefore, it is important to take some measures to further improve the material performance for instance changing the structures, introducing other polymer layers and modifying the surfaces.^[^ ^57^, ^148^ ^]^

### 3.1 Nanofibers in Face Masks and Respirators

Nanofibers are typically <1 µm in diameter.^[^ ^149^, ^150^ ^]^ Common fiber filters in face masks and respirators are usually made of PP fibers with diameters of several tens of microns, and the porosity of the filter is usually 80–90%. Should electret charging not be adopted, the filtration efficiency of microfiber masks would not be adequate to filter fine aerosols based on the structure of the filter layer, in which only the large aerosols can be prevented. In contrast, due to their small diameters, nanofibers possess large surface area‐to‐volume ratios, allowing them to be light while also increasing the probability of aerosol particles being entrapped onto the filter surface composed of nanofibers.^[^ ^151^, ^152^, ^153^ ^]^ This results in an improved filtration efficiency.

The nanofiber filter provides efficient filtration of the particles through the most penetrating particle size (MPPS) at relatively low and acceptable pressure drop.^[^ ^93^ ^]^ There are three main methods to prepare nanofibers for use in filtration media: melt spraying, electrostatic spinning, and multi‐component fiber spinning, the most common technique of which is the “islands‐in‐the sea” method.^[^ ^154^ ^]^ Podgórski et al. showed a linear dependence between the pressure difference across the fiber filter and its thickness.^[^ ^70^ ^]^ The current electrostatic spinning technology has been quite mature, with relatively low cost, and mass production capacity, but it does not have any antiviral or bactericidal functions by itself.

Functional nanofibers, like those modified with amino‐functionalized silica particles or nanodecoys, can trap with charge interactions or proactively identify viruses, and can also be used for manufacturing mask by nanospinning or 3D printing.^[^ ^143^, ^155^, ^156^, ^157^, ^158^ ^]^ Therefore, the filtration performance of these masks can be further improved with those functional modifications in nanofibers.

### 3.2 Introduce Other Polymer Layers

In addition to nanofibers, it can also increase filtration efficiency for fine nanoparticles by introducing other polymer layers and some nanostructures on nanofibers. Other polymer layers can work as substitutes of common filter materials or additional physical barrier, meanwhile they provide electrostatic absorption if adopting electret charging, similar to the principle of multilayer cloth masks. In 2015, Wang et.al fabricated an ultralight binary structured membrane using nylon 6‐polyacrylonitrile nanofiber nets (N6‐PAN NNB) for improving the filtration efficiency to particulate matter of <2.5 µm in diameter (PM2.5).^[^ ^159^ ^]^ This polymer membrane showed high filtration efficiency of up to 99.99%, much better compared to the common commercial masks and respirators. Moreover, polymer materials can be coated on the surfaces of filter fibers or the layers. Recently, a study had achieved the fabrication by coating polypropylene spunbond layers with electrospinning cellulose acetate (CA) and polyvinylidene fluoride (PVDF), both of which can satisfy the standards of N95 filtration performance of National Institute for Occupational Safety and Health (NIOSH).^[^ ^160^ ^]^ This indicated that polymer materials like N6‐PAN NNB have great potential in integrating into masks and respirators to further improve the filtration performance.

## 4 Modifying Masks with Multifunctionality

**Figure 6:**
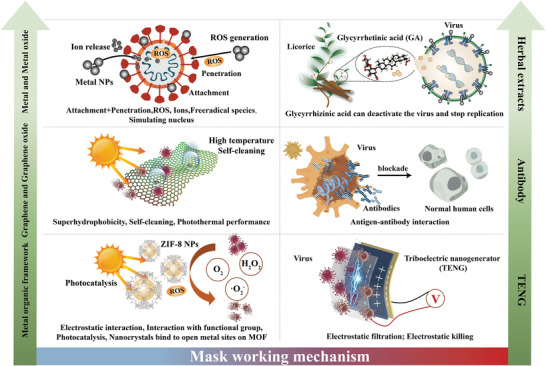
The working mechanisms of multifunctional masks modified with different materials or technologies: a) Metal and metal oxide: 1) Nanoparticles (NPs) attach themselves to the virus, which disrupts the virus from attaching itself onto a potential host cell. 2) NPs produce oxygen, ions and free radical species which are highly reactive. These species then adhere to the membrane walls of microorganisms, reacting with them and potentially destroying the virus's structure and disrupting protein and nucleic acid production. 3) NPs indirectly destroy viruses by activating the immune response of infected cells by stimulating their nuclei – this inhibits the spread of virus. b) Bio‐based or herbal extracts: licorice root is used to fabricate the nanofibers due to its viral inactivation compounds including glycyrrhizin (GL) and glycyrrhetinic acid (GA), which possess an antimicrobial capacity. c) Graphene and graphene oxide: Masks can get benefits that exhibit outstanding performances such as super‐hydrophobicity, self‐cleaning, self‐reporting and excellent photothermal capabilities – addressing limitations found in current ordinary surgical masks. d) Antibody: The use of filters containing ostrich antibodies may be an effective way to prevent virus transmission. e) Metal organic framework (MOF): 1) electrostatic interaction with MOF, 2) interaction with functional groups on MOF and/or polymers, and 3) nanocrystals binding to open metal sites on MOF. f) Triboelectric nanogenerator (TENG): generating electrostatic charges prolong the service time and enhance filtration efficiency while at the same time potentially killing the virus.

As mentioned in Section 1.2, the key layer that enables masks to perform their functions is the filter layer composed of a nonwoven melt blown material. The filter layer is used to filter out fine aerosols that may contain viruses and bacteria. However, as the mask material only captures aerosols and does not actively kill or inactivate pathogens, if there are virus residues on masks, the used masks can become fomites and cause secondary transmission or cross‐infection, particularly when the masks are disposed of inappropriately. Remedying this problem by integrating antiviral components into the filter layer can make the mask more effective, work longer, and less difficult to handle after use. This section reviews the progress of materials and technologies with antiviral properties, the mechanisms which underpin their antiviral function, and their potential to be integrated for use in masks, as shown in **Figure** **6**. Furthermore, this section summarizes the performance of typical antiviral and antibacterial materials (**Table** **1**), and the main mechanisms of typical antiviral and antibacterial materials (**Table** **2**).

**Table 1:** Performance of typical antiviral/antibacterial materials prepared in masks

Types of masks		Preparation	Performance		Reference
			Filtration efficiency	Fitness	
Surgical mask		Nonwoven melt blown and spun bonding	>95% filtration efficiency for aerosol particles	General fit	[79, 81]
N95 level respirators		Nonwoven melt blown and spun bonding	>95% filtration efficiency for aerosol particles	Good fit	[5, 79]
Cloth mask		Homemade using various fabrics	Dependent on structure and materials	General fit	[35, 90]
3D‐Printed mask		Design models and 3D printing	Generally, <95% filtration efficiency for aerosol particles	Good fit but air leakage at interfaces	[132, 140]
Nanofiber mask		Melt spraying, electrostatic spinning or multi‐component fiber spinning	Better filtration efficiency for fine aerosols than common surgical masks and N95 level respirators	General fit	[70, 154]
Metal‐Based particles mask	Au nanoparticles (NPs)	Chemical reduction	92% viral infection reduction after 6 h		[174, 305]
	Ag NPs	Electrochemical	The cell survival rate reaches 98% after the infected cells cultured in 100 ppm Ag NPs for 48h		[183, 305]
	Ag_2_O|AgO NPs	Algae biosynthesized	90% reduction in cytopathic effect (CPE) of HSV‐1 after applying Ag_2_O|AgO NPs and Au NPs		[187]
	Cu NPs	Coating	Under solar illumination, rapidly increase to >70°C and destruct the membrane of nanosized (∼100 nm) virus‐like particles		[188]
	CuO NPs	Surface modification	Five orders of magnitude improvement in killing viruses compared to N95		[190, 305]
	TiO_2_	Sonochemical	Extraordinary antiviral efficiency against NDV at a certain concentration		[192, 305]
Salt‐recrystallization		Natural salt recrystallization	A 100% survival rate of mice exposed to the virus penetrated through the salt‐coated filters		[198]
SiO_2_‐AgNPs		Aerosolize	The average anti‐viral efficiency of the commercial air filter reached ∼92% after coated with the aerosolized SiO_2_‐Ag NPs by a dry aerosol‐coating method		[199]
Graphene‐related	Graphene	Laser‐Induced	The inhibition rate of graphene against bacteria was about 81%; Combined with the photothermal effect, LIG can achieve 99.998% bacterial inactivation efficiency in 10 minutes, and the virucidal efficacy against HCoV‐229E and HCoV‐OC43 can achieve 95% and 97.5% respectively.		[205, 206]
	Graphene Oxide	Oxidation	The inhibition rate of Staphylococcus aureus and Escherichia coli was about 75% and 45%, respectively		[218]
Metal organic framework (MOF)		Chemical	Air filters made with zinc‐imidazolate MOF (ZIF‐8) achieved a photocatalytic killing efficiency of > 99.99% for bacteria within 30 min		[247]
Bio‐based/herbal extracts	Licorice	Extraction	The capture and inhibition properties of licorice root cause rapid inactivation of the virus		[262]
	Herbal Extract Incorporated Nanofiber Fabricated	Electrospinning	With 99.99% filtration efficiency and 99.98% antimicrobial activity against Staphylococcus epidermidis		[273]
Triboelectric nanogenerator (TENG) mask		Implant TENG layers into masks	>95% filtration efficiency		[290, 291, 292]

**Table 2:** Main mechanisms of typical antiviral/antibacterial materials in masks

Types of masks		Main mechanisms	Advantages	Disadvantages	Reference
Metal‐Based particles mask	Main metal nanoparticles	1) Inhibit attachment of the virus. 2) Produce highly reactive oxygen, ions and free radical species. 3) React with microorganisms and potentially destroy the virus structure and disrupt reproduction. 4) Activate the immune response of infected cells by simulating their nucleus.	Details are listed below.	Details are listed below.	[161, 162, 163, 164, 165, 166, 167, 168, 169, 170, 171, 172, 173, 180, 181, 182, 183, 184, 185, 186, 187, 188, 189, 190, 191, 192, 193, 194]
	Au NPs	Inhibit attachment of the virus.	Excellent stability, biocompatibility and bioconjugation.	Expensive.	[170, 171, 172, 173]
	Ag NPs	Inhibit attachment and penetration of virus.	Much cheaper than gold and can be widely used in textiles, medical equipment and wound dressing materials.	Need further study of practical face masks performance.	[180, 181, 182, 183, 184, 185, 186, 187, 188, 189]
	Cu NPs	Destroy the membranes of virus thanks to excellent photoactivity.	Much cheaper than gold and silver	Potential risk of burns under sunlight.	[188]
	CuO NPs	Destroy the integrity of capsid of virus and degrade the whole genome.	Cheap, chemically stable and have shown extensive antibacterial properties.	Need to be further studied.	[189, 190]
	TiO_2_	Destroy the lipid membranes of viruses and block attachment.	Need to be further studied.	Need to be further studied.	[168, 192]
	ZnO	Prevent entry of viruses.	Need to be further studied.	Need to be further studied.	[170, 193, 194]
Salt‐recrystallization		Recrystallization causes the jagged salt crystals pierce the virus membrane and kill it.	Can be safely used and preserved or reused for a long term under such high humidity and temperature condition, low‐cost and public protection.	Need to be further studied.	[198]
Masks based on Graphene‐related materials	Graphene	1) Kill viruses by photothermal effect. 2) Inhibit attachment of bacteria. 3) Hydrophobic LIG can potentially induce dehydration for bacteria.	Outstanding superhydrophobicity, self‐cleaning and self‐reporting capabilities and excellent photothermal performances.	Potential risk of burns under sunlight.	[205, 206, 207, 208, 209, 210, 211, 212, 213, 214, 215, 216, 217]
	Graphene Oxide	1) Cut the outer membranes of bacteria as well as exert oxidative stresses on the bacteria. 2) Inhibit attachment and entry of viruses.			[200, 210, 218, 219, 220, 221, 222]
Masks based on MOF		Specifically, zinc‐imidazolate MOF (ZIF‐8) can kill virus via reactive oxygen species (ROS) released by photocatalytic effect.	Fiber surfaces coated with electro‐thermally stable MOFs or ZIF‐8 nanofibers can eliminate bacteria and viruses after use.	Need to be further studied.	[238, 239, 240, 241, 242, 243, 244, 247]
Bio‐based/herbal extracts	Licorice extracts	Prevent viruses from replicating or inactivate them.	Low toxicity, high antimicrobial activity, mild environmental effect and low cost.	Durability remains a concern.	[258, 259, 260, 261]
	Some other herbal Extracts	The contained flavonoids kill microorganisms by disrupting cell membrane function and inhibiting DNA cyclase.			[263, 264]
TENG mask		Absorb fine aerosols and kill viruses by electric high voltage.	Prolong mask's lifespan.	The efficacy against SARS‐CoV‐2 and the durability need to be further studied.	[287, 288, 289, 290, 291, 292]
Antibody technology		React specifically with a certain antigen such as a virus or bacteria and destroy it.	Has been developed to an improved functional nonwoven air filter.	The efficacy against SARS‐CoV‐2 and the durability need to be further studied.	[293]

### 4.1 Metal and Metal Oxide

**Figure 7:**
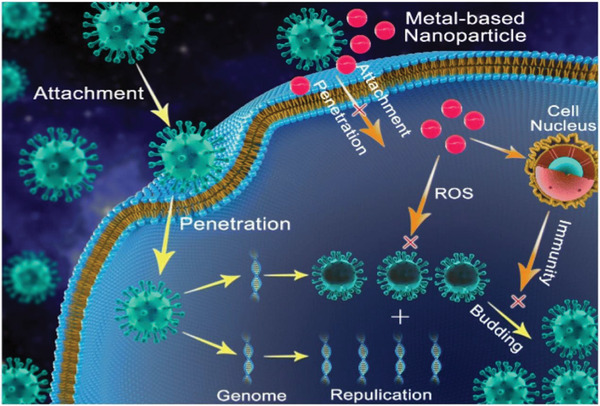
A schematic representation of the antiviral mechanism of metal‐based nanoparticles. Reproduced with permission.^[^ ^322^ ^]^ Copyright 2020, Springer Nature.

Nanoparticles based on metallic elements, i.e., metal‐based nanoparticles are small and contain large specific surface areas. This gives them novel physiochemical properties, main of which is the ability to disrupt the functions of viruses, bacteria, and other microorganisms, potentially killing them. As such, metal and metal oxide nanoparticles have been applied to imbue antiviral properties on materials, with the most commonly metals being gold (Au), silver (Ag), zinc (Zn), copper (Cu), and titanium (Ti).^[^ ^161^, ^162^, ^163^, ^164^, ^165^, ^166^, ^167^, ^168^, ^169^, ^170^, ^171^, ^172^ ^]^ As shown in **Figure** **7**, four major interaction phases have been identified as to how these metal nanoparticles exhibit antiviral properties: 1) the nanoparticles attach themselves to the virus, which disrupts the virus from attaching it onto a potential host cell; 2) the air flow causes slight ionization of the metal nanoparticle layer on the surface. Under the synergistic action of adsorption and diffusion, a microenvironment with metal nanoparticle is formed. When the metal nanoparticle contacts bacteria or viruses, it can rapidly oxidize the core material of bacteria or viruses by stimulating the generation of reactive oxygen species to realize the inactivation effect; 3) When the bacteria or viruses contact with the metal nanoparticle clusters, the metal nanoparticles adhere to the membrane walls of the microorganisms, and the denaturation and deactivation of the specific proteins on the surface of the bacteria or viruses are caused, and then the apoptosis occurs; 4) they indirectly destroy the virus through activating the immune response of infected cells by simulating their nucleus – this inhibits the spread of the virus.

**Figure 8:**
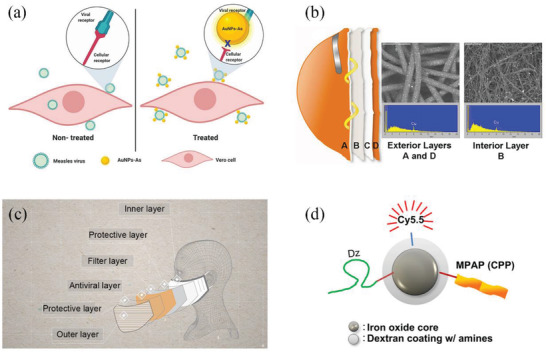
Some anti‐microbial applications of metals and its derivatives. a) The antiviral schematic diagram of gold nanoparticles. Reproduced under the terms of the Creative Commons Attribution 4.0 International License.^[^ ^174^ ^]^ Copyright 2019, The Authors. Licensee MDPI, Basel, Switzerland. b) N95 mask treated with CuO NPs has antiviral property to kill viruses retained by the mask without changing the physical barrier performance. Reproduced under the terms of the Creative Commons Attribution License.^[^ ^190^ ^]^ Copyright 2010, Borkow et al. c) The structure diagram of CuMask+ provided by the Hong Kong Government. Reproduced with permission.^[^ ^191^ ^]^ Copyright 2020, The Hong Kong Research Institute of Textiles and Apparel Limited (HKRITA). d) The working mechanism of a multifunctional iron oxide nanoparticle for DNAzyme delivery (Dz, DNAzyme; MPAP, myristoylated polyarginine peptide; Cy5.5, fluorescent dye; CPP, cell‐penetrating peptide). Reproduced with permission.^[^ ^197^ ^]^ Copyright 2012, Elsevier.

Gold nanoparticles (Au NPs) cause the suppression of the virus by blocking site where the virus particles can bind to potential host cells, thereby inhibiting the attachment or entry of the virus and controlling the transmission of the virus from cell to cell. Au NPs have excellent stability, biocompatibility, and the capacity to bind to biological ligands (bioconjugation), which are therefore closely related to the application of antiviral materials.^[^ ^170^, ^171^, ^173^ ^]^ Mayra et al. made Au NPs whose average particle size is 10 nm through chemical reduction and used plant extracts as a reducing agent. It showed that the gold nanoparticles significantly reduced measles virus infection (by 92% after 6 hours of interaction).^[^ ^174^ ^]^ The viricidal effect of gold nanoparticles may be due to Au NP's interaction with the measles virus receptor to inhibit attachment to host cells and avoid viral infection, as shown in **Figure** **8**a. Baram–Pinto et al. described the application of Au NPs coated with mercaptoethylsulfonate (Au‐MES NPs) as an efficient inhibitor of herpes simplex virus type 1 (HSV‐1) infection because they can mimic the cell membrane receptor heparin sulfate. Their study suggested that Au‐MES NPs interferes with the viral attachment, entry, and cell‐to‐cell transmission, so they can prevent the viral infection.^[^ ^173^ ^]^ Despite their high efficacies in killing pathogens, gold is an expensive metal, thus making it impractical for use in PPEs.

Silver is a much cheaper alternative compared to gold and has been used as a classic antimicrobial substance. Silver and its derivatives are keeping in high demand due to their superior antimicrobial properties, mainly silver nanoparticles (Ag NPs), silver oxide (Ag_2_O NPs), silver monoxide (AgO NPs), which have been widely used in textiles, medical equipment and wound dressing materials.^[^ ^175^, ^176^, ^177^, ^178^, ^179^ ^]^ A study showed that >99% of *Escherichia coli* samples were eliminated when exposed to silk fibers decorated with Ag NPs.^[^ ^180^ ^]^ Ag NPs could directly interact with the outer layer of viruses, and they can inhibit them from attaching to and infiltrating into the host cell. Accelerated rate of Ag^+^ release from Ag NPs was found to be responsible for an increase interaction with the subcellular organelles of bacteria.^[^ ^167^ ^]^ In addition, Tang et al. also demonstrated through depositing Ag NPs on silk as surface coating that Ag NPs is effective against pathogens, which is attributed to their ability to produce the surface plasmon resonance effect, and it can be visually demonstrated with changes in color.^[^ ^181^ ^]^ The average size of Ag NPs is a vital parameter affecting their antiviral performance. Then they demonstrated that the ion release rates and antimicrobial properties depend on the size of Ag NPs.^[^ ^182^ ^]^ Krzyzowska et al. prepared Ag NPs whose average particle size is 33 nm using chemical reduction method,^[^ ^180^ ^]^ and Huy et al. obtained Ag NPs whose average particle size is 7.1 nm using electrochemical method,^[^ ^183^ ^]^ so that the small average particle size ensures that they can interact effectively with viruses which also have a small size. Huy et al. also reported that Ag NPs can neutralize poliovirus (25–30 nm) by destroying its protein molecules, so they can inhibit the attachment to host cells. Besides, the average size of Ag NPs can be controlled by adding surfactants to the synthetic method.^[^ ^180^, ^183^ ^]^ Since average particle size has a strong effect on the efficacy to neutralize viruses, surfactants such as plant polyphenols, citric acid, and PVP are often applied when processing Ag NPs to enhance their anti‐viral properties.^[^ ^184^, ^185^, ^186^ ^]^ Mostafa et al. proved that a 90% reduction in cytopathic effect (CPE) of Herps Simplex virus (HSV‐1) was achieved by utilizing Ag_2_O|AgO NPs (size: 14.42–48.97 nm) and Au NPs (size: 15.60–77.13 nm). And the reduction rate (49.23%) with Ag_2_O|AgO NPs is higher than that of Au‐NPs (42.75%).^[^ ^187^ ^]^ So they showed the efficiency when applying nanoparticles with both Ag_2_O|AgO NPs and Au NPs to act as reducing and inhibitory agents for the HSV‐1. However, the results and evidence to date on the antiviral properties of practical face masks decorated with Ag NPs, Ag_2_O NPs, AgO NPs are scant and it needs to be addressed in a broader field.

Cu NPs, copper oxide (CuO) and copper oxide nanoparticles (CuO NPs) can destroy the integrity of viruses and destroy their genomes. A study showed a nanocoating mixture of shellac and Cu NPs assisted with two‐channel spray was applied to a nonwoven surgical mask, and it can increase the surface's hydrophobicity and ability to repel water droplets.^[^ ^188^ ^]^ Then the surfaces show excellent photoactivity (both photocatalytic and photothermal properties) with antimicrobial properties, which make the masks reusable and able to self‐sterilize. Under sunlight, the temperature of this photosensitive antiviral masks (PAM) rise quickly to >70 °C, which produces high concentration of free radicals and they can destroy the membranes of nanoscale (≈100 nm) virus‐like particles. This can make the mask able to self‐clean and reusable.^[^ ^188^ ^]^ Ahmad et al. showed that CuO NPs release copper ions that can act as a catalyst to create reactive oxygen species (ROS), thus destroying the integrity of capsid of herpes simplex virus (HSV) and degrading the whole genome.^[^ ^189^ ^]^ Gadi et al. reported that by immersing N95 mask in CuO NPs to prepare antiviral respiratory protective masks, CuO NPs attached to the mask could kill viruses retained in the mask without changing the physical barrier performance, as shown in Figure 8b.^[^ ^190^ ^]^ Indeed, this study showed that N95 masks treated with CuO NPs displayed better antiviral performance compared to untreated masks by five orders of magnitude. Furthermore, CuO NPs are cheap, chemically stable, and have shown extensive antibacterial properties, making it a popular choice in the production of materials that require antiviral functions. For example, in 2020, Hong Kong Government provided the copper‐core anti‐epidemic mask (CuMask+) for its citizens for free, which was developed by The Hong Kong Research Institute of Textiles and Apparel Limited (HKRITA),^[^ ^191^ ^]^ as shown in Figure 8c.

In addition to these materials, studies on anti‐microbial performances have also been conducted on nanomaterials containing other metallic elements. Titanium oxide (TiO_2_) has been successfully evaluated in microbiological field. The TiO_2_ nanoparticles (TiO_2_ NPs) sample prepared by Sara et al. showed excellent antiviral performance against Newcastle Disease Virus (NDV), and its inhibition mechanism relies on destroying the lipid membranes of viruses and blocking viruses from attaching to host cells.^[^ ^192^ ^]^ In most cases, TiO_2_ are combined with inorganic metal, such as copper (Cu), silver (Ag), and manganese (Mn), 2D materials (e.g., MXenes, MOF, and graphene), and nonmetallic, including Fluorine (F), calcium (Ca), and phosphorus (P), to interact with charge transfer mechanisms, crystallinity, surface porosity, and microbial disinfection efficiency.^[^ ^168^ ^]^ Antimicrobial activity of TiO_2_ coating is targeted for the most critical pathogenic microorganisms including methicillin‐resistant *Staphylococcus aureus*, *E. coli*, *Bacillus subtilis*, *Pseudomonas aeruginosa*, *Staphylococcus aureus*, *Legionella pneumophila*, *Streptococcus mutans*, Bacteriophage H1N1, T2, vesicular stomatitis virus, HCoV‐NL63, bovine coronavirus.^[^ ^168^ ^]^

Zinc oxide nanoparticles (ZnO NPs) exhibit extraordinary microbial activity against a variety of microorganisms, such as viruses.^[^ ^170^ ^]^ Yogendra et al. demonstrated that for HSV, ZnO NPs use a mechanism of capturing the virus particles, thereby disabling them from getting to the host cell in the first place.^[^ ^193^ ^]^ Zinc oxide micro‐nano structures (ZnO‐MNSS) which is partially negatively charged effectively traps virus through a novel viral inhibition mechanism that prevents them from entering human corneal fibroblasts, the target cells of HSV‐1 infection. Besides, zinc oxide tetrapods (ZnOTs) can efficiently prevent herpes simplex virus type 2 (HSV‐2) from entering target cells and prevent virus transmission.^[^ ^194^ ^]^

The antiviral properties of other metals and metal oxides have also been investigated, such as gallium, tin oxide and iron oxide (shown in Figure 8d), which may be embedded in masks for use in PPE.^[^ ^195^, ^196^, ^197^ ^]^

### 4.2 Antiviral Chemical Compound

The most common antiviral chemical compound in our life is salt, which is usually used to preserve food through inactivating microorganisms or inhibiting microbial growth. Quan et al. proved that the filtration system relying on salt‐recrystallization can provide high filtration efficiency and inactivate many adsorbed virus subtypes with success.^[^ ^198^ ^]^ Furthermore, they noted that the high humidity and temperature did not affect the stability of the salt coating, which indicates that the salt coating can be safely used and preserved or reused for a long term under such environmental conditions. The destruction mechanism to virus only depends on the simple and natural process of salt‐recrystallization, based on the unstable effect of salt crystal growth with the increasing concentration when it is drying. The idea could be directly applied to the existing technologies in a wider range to acquire universal personal, low‐cost and public protection, including face masks and air filters, against airborne pathogens. Hence, it can be predicted that the salt‐recrystallization filtration system could contribute to global health through providing effective and reliable masks to prevent COVID‐19 from spreading.

Moreover, other chemical compounds also possess the antiviral ability. Park et al. produced an antiviral material containing SiO_2_‐Ag NPs and coated it on commercial air filters.^[^ ^199^ ^]^ The modified filter was then tested for aerosol‐resistance to phage. At a certain velocity of medium, the filtration efficiency, and antiviral efficiency were improved with the increase of SiO_2_‐AgNPs concentration.

### 4.3 Graphene‐Related Materials

Graphene has indicated a promising potential in the control of the epidemiological spread of the disease.^[^ ^200^, ^201^, ^202^, ^203^, ^204^, ^205^, ^206^ ^]^ Graphene's substantial surface area‐to‐volume ratio provides the highest ligand contact area which can interact with the charged residue of virions to block microorganisms.^[^ ^207^, ^208^, ^209^ ^]^ It is theorized that laser induced graphene (LIG) irreversibly damages bacteria when both are brought into contact together.^[^ ^210^ ^]^ In addition, it has been reported that the rough surface of graphene can prevent bacterial cells from attaching to host cells, thereby preventing their proliferation.^[^ ^211^ ^]^ Besides, interaction between sharp edges of graphene is also possibly related to LIG's bactericidal ability.^[^ ^212^ ^]^ Furthermore, oxygen containing functional groups, including ‐OH and ‐COOH, are present in the hydrophilic graphene, resulting in a potential charge transfer between the outer layer of bacteria and LIG, which may lead to an intracellular material loss for the bacteria.^[^ ^213^ ^]^ It has also been shown that the structure of LIG exerts both chemical and physical stress upon bacteria, which further improves its bactericidal properties. As for hydrophobic LIG, its hydrophobicity can potentially induce dehydration for a bacterium, leading to its inhibition.^[^ ^213^, ^214^ ^]^

**Figure 9:**
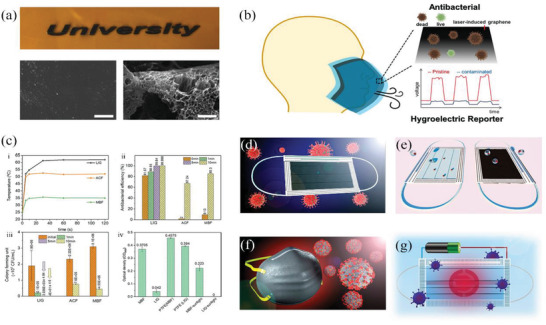
Masks in graphene‐related materials. a) Conversion of polyimide (PI) to LIG: Optical image of PI and LIG, SEM image of PI and LIG (Scale bar is 10 µm).^[^ ^205^ ^]^ b) Self‐reporting and photothermally enhanced rapid bacterial killing on a laser‐induced graphene mask.^[^ ^205^ ^]^ c) Enhanced antibacterial efficacy using the photothermal effect.^[^ ^205^ ^]^ LIG face mask with d) photothermal ability to kill viruses and e) superhydrophobic ability.^[^ ^215^ ^]^ f) Superhydrophobic graphene N95 respirator with self‐decontaminating property.^[^ ^216^ ^]^ g) Reusable electrothermal graphene mask with self‐sterilization property.^[^ ^225^ ^]^ a‐c) Adapted with permission.^[^ ^205^ ^]^ Copyright 2020, American Chemical Society. d‐e) Adapted with permission.^[^ ^215^ ^]^ Copyright 2020, American Chemical Society. f) Reproduced with permission.^[^ ^216^ ^]^ Copyright 2020, American Chemical Society. g) Adapted with permission.^[^ ^225^ ^]^ Copyright 2020, American Chemical Society.

Recent studies have reported that reusable and recyclable graphene masks are highly effective against COVID‐19 with the added benefits that these masks demonstrate outstanding superhydrophobicity, have both self‐cleaning and self‐reporting capabilities, as well as showcasing excellent photothermal performances, thereby addressing limitations that currently exists in ordinary surgical masks.^[^ ^205^, ^214^, ^215^, ^216^ ^]^ As shown in **Figure** **9**, graphene‐related materials have been reported to show superhydrophobic characteristics and antimicrobial activity on the surface‐level of the material. Zhong et al.^[^ ^215^ ^]^ deposited a lamina layer of graphene onto standard commercial woven face masks using the technique of dual‐mode laser‐induced transfer. These graphene‐layered masks exhibited two key properties that render them effectively against viruses transferred via droplets – outstanding hydrophobicity and photothermal properties, as shown in Figure 9d,e. The superhydrophobic graphene film addresses the issue of droplet accumulation by directly rejecting incoming droplets. The graphene‐surface provides a protective layer wherein incoming water‐based droplets are simply repelled back due to graphene's superhydrophobicity, thereby eliminating droplet accumulation. This layer significantly improves the self‐cleaning ability of the masks from droplets generated by both outsiders and the user as they exhale. As the droplets no longer accumulate on the masks, the risk of secondary transmission caused by improper use and disposal of masks can be significantly reduced.^[^ ^214^, ^215^ ^]^ The second key property of graphene film surface is its photothermal properties – the surface of the graphene‐film can reach up to temperatures of 80 °C without destruction when it is subjected to sunlight irradiation. At these elevated temperatures, most viruses cannot survive. For instance, report has shown that SARS‐CoV‐2 can be inactivated at elevated temperatures, which the graphene‐layer can achieve within minutes of being subjected to sunlight.^[^ ^217^ ^]^ Thus, the photothermal property of the graphene coating gives the mask a self‐sterilizing ability as well. With regards to bacteria, the inhibition rate of graphene alone against bacteria is already ≈81%, which rises to 99.998% bacterial‐killing efficiency in 10 min when synergized with the photothermal effect.^[^ ^205^ ^]^ Then, it is reported that the virucidal efficacy of the hydrophobic LIG against HCoV‐229E and HCoV‐OC43 can reach 95% and 97.5% respectively under low solar irradiation.^[^ ^206^ ^]^ These two properties of superhydrophobicity and excellent photothermal effects allow the graphene‐layered masks to both be reusable and recyclable and can have significant medical applications.

Furthermore, studies have indicated that the ultra‐thin surface of graphene oxide (GO) can both destroy the outer membranes of bacteria as well as exert oxidative stresses on the bacteria – the combined effects of which can weaken or even kill bacteria.^[^ ^210^, ^218^, ^219^, ^220^, ^221^ ^]^ More importantly, GO has also shown anti‐viral capabilities.^[^ ^200^ ^]^ The unique structure and ionic charge of GO can suppress receptors and destroy viral structures before the virus enters a host cell, neutralizing the virus.^[^ ^222^ ^]^

**Figure 10:**
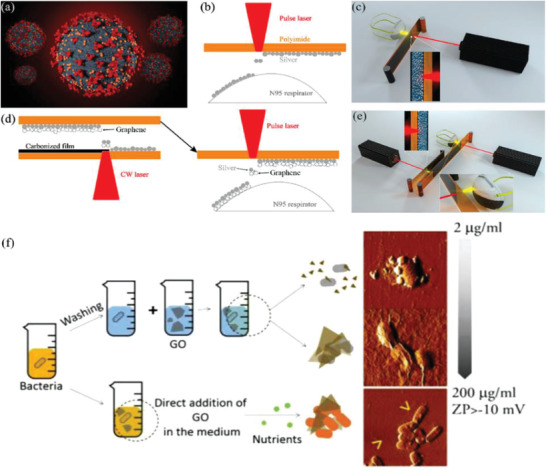
The fabrication processes of LIG‐Ag NPs mask. a) Illustration of the SARS‐COV‐2 virus, b) M1 laser printing strategy, c) Illustration of the setup of the M1 laser printing method, d) M2 laser two‐step laser printing strategy, e) Illustration of the setup of the M2 laser printing method. a‐e) Reproduced with permission.^[^ ^216^ ^]^ Copyright 2020, American Chemical Society. f) The antibacterial or growth‐promoting effects on *Staphylococcus aureus* and *Escherichia coli*. Adapted with permission.^[^ ^218^ ^]^ Copyright 2017, American Chemical Society.

To further enhance GO's efficacy, silver particles can be fixed onto GO (GO‐Ag) flakes to further inhibit the infectivity of both non‐enveloped and enveloped viruses.^[^ ^223^, ^224^ ^]^ For non‐enveloped viruses, the GO flakes act as a supporting material for the antiviral Ag particles without any antiviral capabilities themselves. For enveloped viruses, GO flakes serve a dual purpose: they help Ag particles disperse evenly, and they also act as inhibitory agents against viral infections. Furthermore, masks coated with plasma Ag NPs and LIG can use solar energy alone to achieve plasma photothermal decontamination, as shown in **Figure** **10**. While these functional masks exhibit excellent superhydrophobic and photothermal properties as previously mentioned, the incorporation of Ag creates the added benefit of silver ions being released into micro‐organisms into the exhaled droplet. This creates a synergistic effect that provides even stronger protection against the SARS‐CoV‐2 virus – plasma heating can increase surface temperature to >80 °C within 1 min under sunlight, the superhydrophobic feature prevents droplet‐accumulation of the mask or respirator surface, and the silver nanoparticles can disinfect any droplets exhaled by the user before it reaches the environment.^[^ ^216^ ^]^

However, the photothermal performance of the masks described above may not be controlled or regulated by the user, which poses the risk of high mask temperatures while the mask is being worn. In view of this disadvantage, Shan et al. developed an electrothermal graphene mask, which also showed an excellent self‐sterilization performance, as shown in Figure 9g. Despite only using a low voltage of 3 V, this mask can achieve a high temperature above 80 °C within a very short duration.^[^ ^225^ ^]^ Although current graphene mask technologies are not yet mature and still have some flaws that still require further investigation, the graphene‐based substances have demonstrated many excellent properties and potential for many applications.

### 4.4 Metal Organic Framework

**Figure 11:**
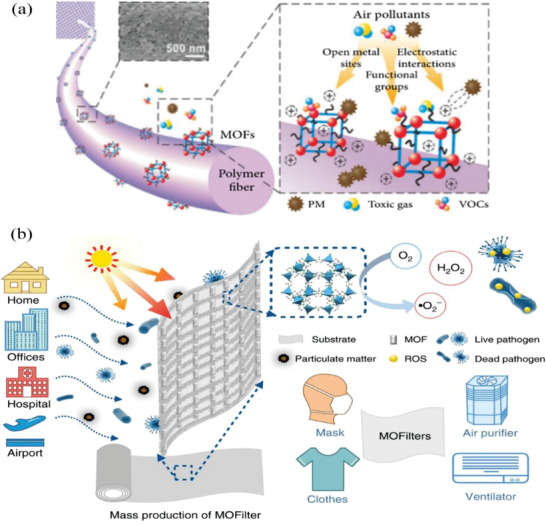
The mechanism diagram of MOF cleaning air. a) The surface of the MOF/polymer composite fiber. Reproduced under the terms of the Creative Commons Attribution 4.0 International License.^[^ ^244^ ^]^ Copyright 2020 Ming Hui Chua et al. Exclusive Licensee Science and Technology Review Publishing House. b) The schematic of a metal‐organic framework (MOF)‐based filter for integrated air cleaning. Adapted under the terms of the Creative Commons Attribution 4.0 International License.^[^ ^247^ ^]^ Copyright 20219, Springer Nature.

Most masks, whether woven or non‐woven, rely on dense fibers to resist harmful microorganisms under high air resistance. These fibers only acts as physical barriers or adhesion sites for droplets,^[^ ^226^, ^227^, ^228^, ^229^, ^230^ ^]^ which only allow bacteria, fungi and viruses within these droplets to adhere to the filter surface rather than being completely eradicated.^[^ ^228^, ^229^ ^]^ Eventually, with the aid of the accumulation of other organic pollutants that act as a nutrition‐source, the filter becomes a hotbed for pathogenic microorganisms, thereby creating the potential for secondary viral airborne transmission.^[^ ^231^ ^]^ At the same time, accumulation of organic matter may reduce gas permeability, hence reducing wearer comfort, among other effects. As such, the photocatalytic bactericidal characteristics of metal organic frameworks (MOF) have great potential to overcome these aforementioned disadvantages in most conventional masks that utilize dense fibers.^[^ ^232^, ^233^, ^234^, ^235^, ^236^, ^237^ ^]^ Among the emerging classes of antimicrobial agents being developed, MOFs are predominant thanks to the uniform distribution of metal active sites, high surface area and modifiable porous structures.^[^ ^238^, ^239^, ^240^, ^241^ ^]^ Studies have recognized that the main antibacterial mechanisms of MOFs depend on the intrinsic biological properties of the metal ions and the antimicrobial properties of organic ligands.^[^ ^238^, ^242^, ^243^ ^]^ Nevertheless, MOF‐based filters can capture particulate contaminants through three primary mechanisms: 1) electrostatic interaction between the contaminant and the MOF, 2) contaminant interaction with functional groups on the MOF, and 3) nanocrystal‐binding with open metal sites on the MOF, as shown in **Figure** **11**a.^[^ ^244^ ^]^

Recently, rapid progress has been presented in the study of both antimicrobial properties and the antimicrobial applications of MOF substances and related composites for air filters and masks.^[^ ^238^, ^243^, ^245^, ^246^ ^]^ Specifically, Li et al. have shown that zinc‐imidazolate MOF (ZIF‐8) can nearly completely inactivate E. coli with a >99.9999% inactivation efficiency. The study also showed that photoelectrons which is trapped in Zn^+^ centers within ZIF‐8 were responsible for reducing molecular oxygen into reactive oxygen species (ROS) through ligand to metal charge transfer (LMCT), and this is the main disinfection pathway that leads to the photocatalytic disinfection ability of ZIF‐8.^[^ ^247^ ^]^ The Zn^+^ ions charge capture center of the Zn^+^ ions can be generated on the surface of the MOF through LMCT, and O_2_ can be effectively activated to form •O^2−^ and other related ROS, such as H_2_O_2_, as shown in Figure 11b. Hence, the actual biocidal property of ZIF‐8 mostly arises from the production of ROS instead of the release of Zn^2+^. ZIF‐8 has an outstanding photocatalytic antibacterial performance, far better than that of traditional semiconductors such as TiO_2_ and ZnO. In addition, ZIF‐8 filters integrate the particulate matter (PM) filtration functions with sterilization functions. Results show that the air filter made of ZIF‐8 exhibited excellent performance in integrated pollution control with a photocatalytic killing efficiency of over 99.99% for bacteria in air within 30 min.^[^ ^247^ ^]^ This work reveals the photocatalytic antimicrobial role of MOF and provides valuable and significant insights into potential antimicrobial applications of MOF in air filters, masks and disinfectants.

Furthermore, fiber surfaces coated with electro‐thermally stable MOFs or ZIF‐8 nanofibers can be subjected to high temperature, ultraviolet radiation and hydrogen peroxide treatment to eliminate bacteria and viruses, thereby maintaining safety after use and reducing the risk of secondary transmission if integrated into masks.^[^ ^238^, ^242^, ^243^ ^]^ This will aid in the design of new porous solid materials with photocatalytic anti‐microbial functions for use in mask production to protect health workers and the general public.

### 4.5 Bio‐Based Substances or Herbal Extracts

**Figure 12:**
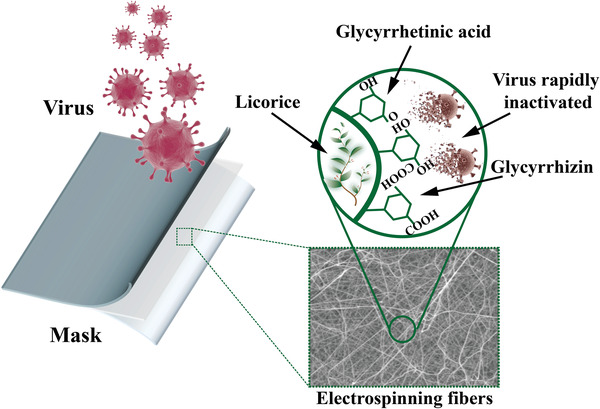
Schematic illustration of inactivation mechanism to viruses of using an antiviral mask with herbal extracts.^[^ ^262^ ^]^

Herbs have been used in medicine since ancient times, including for the purposes of treating wounds which inadvertently protected them against microbes long before the emergence of germ theory.^[^ ^248^, ^249^, ^250^, ^251^ ^]^ Studies have shown that herbal extracts are efficient against viruses including human immunodeficiency virus (HIV), respiratory syncytial virus (RSV), and SARS‐COV, all of which can cause severe pneumonia.^[^ ^252^, ^253^, ^254^, ^255^, ^256^, ^257^ ^]^ In particular, licorice extracts such as glycyrrhizin (GL) and glycyrrhetinic acid (GA) have been demonstrated to be able to destroy biomolecules and possess antiviral properties, primarily by either preventing viruses from replicating or by inactivating them altogether,^[^ ^258^, ^259^, ^260^, ^261^ ^]^ as shown in **Figure** **12**. Based on this antiviral property, licorice can be used to produce bio‐based masks to inactivate SARS‐COV‐2 and to prevent the spread of COVID‐19. Virus‐laden droplets are locked onto the extract reagent, after which they are quickly opened through hydrophilic action, thereby leading to the virus being exposed. Chowdhury et al. combined licorice root extracts containing GA and GL with the electrostatic spinning process to manufacture a licorice root nanofiber membrane, which can be used as the filter layer in the fabrication of antiviral masks. In the licorice root membrane, the nanofiber had diameters ranging from 15 to 30 µm with random porosity and direction.^[^ ^262^ ^]^

In general, the antimicrobial performance of herbal extracts is commonly attributed to the flavonoids that they may contain, which can kill microorganisms by disrupting cell membrane function and inhibiting deoxyribonucleic acid (DNA) cyclase.^[^ ^263^, ^264^ ^]^ Besides licorice extracts, it was also found that some herbal plants with antimicrobial properties, such as sage, garlic, oregano, fennel, etc., can reduce some symptoms of infectious individuals and may prevent the transmission of COVID‐19.^[^ ^265^, ^266^, ^267^, ^268^, ^269^ ^]^ In addition, Sim et al. developed an Activated Carbon Fiber (ACF) filter incorporated with Sophora Flavescens, and they reported that the ACF filter showed an antibacterial efficiency >90%.^[^ ^270^ ^]^ However, the high load of herbal extracts in filters may result in a significantly increased pressure drop.^[^ ^270^, ^271^, ^272^ ^]^ To solve this problem, Choi et al. made antibacterial nanofiber membranes that were prepared by electrospinning a solution of polyvinylpyrrolidone (PVP).^[^ ^273^ ^]^ Because the antimicrobial components are uniformly distributed in polymer nanofibers, the hybrid nanofiber filter paper was able to obtain an antimicrobial activity (99.98%) against S. epidermidis and an excellent filtration efficiency (99.99%).

In summary, the nanofiber membrane prepared by combining herbal extracts with electrostatic spinning technology offers an effective antiviral and antibacterial material that can be potentially used in protective masks. Indeed, antimicrobial herbal extracts, and bio‐based technologies from natural substances have been broadly investigated as antimicrobials for masks and air filters because of their low toxicity, high antimicrobial activity, mild environmental effects, and low cost.^[^ ^274^, ^275^ ^]^ However, for practical applications, the antimicrobial activity of herbal extracts may be affected or even degraded by the oxidation process that occurs naturally via exposure to air or temperature changes overtime, so its durability remains a concern that needs to be addressed.^[^ ^263^, ^276^, ^277^ ^]^

### 4.6 Integrated Triboelectric Nanogenerator

Since the main filtration mechanism in masks involves the electrostatic attraction of electret charging in the middle filter layer and these electrostatic charges are easy to disappear, most masks have a short effective lifespan. Thus, studies have been proposed to improve the conditions of electret charging and hence the longevity of mask filters. Several studies have focused on triboelectric nanogenerators (TENGs), which were originally connected to wearable fabrics in the textile industry.^[^ ^278^, ^279^, ^280^, ^281^ ^]^ TENG has the capability of converting mechanical energy from all kinds of mechanical movements into electricity through triboelectrification and electrostatic induction effect, which has great potential in a great variety of practical applications.^[^ ^282^ ^]^ But recently TENGs have also attracted significant research attention due to their potential application to face masks and respirators in order to prolong service time and enhance filtration efficiency,^[^ ^283^, ^284^, ^285^, ^286^, ^287^, ^288^ ^]^ as shown in Figure 6f. This application has become even more pertinent during the COVID‐19 pandemic.

**Figure 13:**
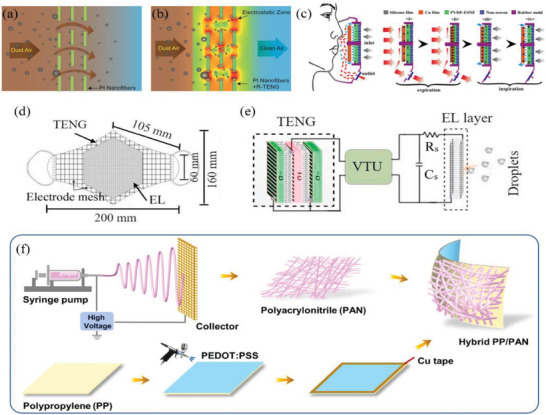
The schematic illustration of different face masks integrated with triboelectric nanogenerator (TENG). a,b) show the filtration performance before and after applying rotating triboelectric nanogenerator in air filter by Gu et al., respectively. a,b) Adapted with permission.^[^ ^289^ ^]^ Copyright 2017, American Chemical Society. c) The structure of respiratory triboelectric nanogenerator for the self‐powered electrostatic absorption face mask by Liu et al. Adapted with permission.^[^ ^290^ ^]^ Copyright 2018, American Chemical Society. d) The proposed design of triboelectric self‐powered mask and e) The diagram of face mask with the TENG by Ghatak et al. d‐e) Reproduced with permission.^[^ ^292^ ^]^ Copyright 2021, Elsevier. f) Schematic illustration of the fabrication process of a new nano/micro fibrous hybrid mask. Reproduced with permission.^[^ ^287^ ^]^ Copyright 2021, Elsevier.

In 2017, Gu et al. utilized a rotating triboelectric nanogenerator (R‐TENG) to devise an air filter using polyimide (PI) nanofiber for removing PM, and it was shown that this set‐up significantly enhanced filtration efficiency of PM, especially for particles with diameters <100 nm, as shown in **Figure** **13**a,b.^[^ ^289^ ^]^ However, the air filter attained its best filtration efficiency of 90.6% only for PM of about 33.4 nm in diameter, which does not meet the requirements for surgical masks and N95 level respirators. Furthermore, Liu et al. developed a self‐powered and long‐lasting electrostatic adsorption mask, which combined the poly‐electrospun nanofiber film with a TENG, as shown in Figure 13c.^[^ ^290^ ^]^ After being worn for 4 h and even after a 30‐day interval, this face mask still showed a high filtration efficiency of 99.2 wt% for particle sizes of 1.0 µm and above and a filtration efficiency of >86.9 wt% for particles of sizes 0.5 µm and below. Moreover, Zhang et al. was able to design a TENG surgical mask that can achieve a filtration efficiency >95% for particles with size of 0.3–10 µm.^[^ ^291^ ^]^ Recently, Wang et al. fabricated a new type of medical mask which replaced the PP layer with polyvinyl alcohol (PVA) and possessed good self‐charging and charge retention ability under an environment of 95% relative humidity.^[^ ^288^ ^]^ This mask was able to better generate short circuit current and also showed a higher static dissipation rate compared to PP‐based medical mask. Moreover, it was proposed that face masks can be combined with TENG to generate electric high power and thereby kill viruses, as demonstrated in Figure 13d,e.^[^ ^292^ ^]^

All these studies have demonstrated that the incorporation of TENG can significantly enhance the filtration efficiency of a face mask to over 95%, and these studies also exhibited the great application‐potential of TENG. However, it is necessary that pre‐existing designs are firstly completed and their practical applications are assessed for them to meet the standards for commercial face masks and respirators.

### 4.7 Antibody Technology

An antibody is a substance that can react specifically with a certain antigen such as a virus or bacterium in order to destroy it, and this is also the mechanism of the human immune system and most vaccines, as shown in Figure 6d. Kamiyama et al. developed an improved functional nonwoven air filter that was imbued with antibodies against the avian influenza H5N1 virus.^[^ ^293^ ^]^ Based on the antigen‐antibody interactions, the resulting filters were found to inactivate viruses trapped in them. The results suggested that the use of filters containing ostrich antibodies is possibly an efficient way to prevent H5N1 transmission. However, this study was limited in that the filters were only tested on birds, with the result that none of the birds in the box with the antibody filter died. Therefore, this method requires further study to determine its efficacy against other viruses and also its performance under changing environmental conditions, such as when the filter is transported, stored and used. Even so, it is expected that when these filters modified with antibodies are used in masks, the resulting masks will also possess antiviral properties. Therefore, the antibody technology has the potential to be applied as a preventive measure against COVID‐19 and therefore warrants further study.

### 4.8 Quantum Dots

Quantum dots (QDs) are semiconductor nanocrystals with special electronic and optical properties which highly depend on size^[^ ^294^ ^]^; and the use of QDs as an antiviral agent has been demonstrated in different ways.^[^ ^170^, ^171^ ^]^ Du et al. have studied the antiviral performance of glutathione (GSH) capped CdTe QDs utilizing pseudorabies virus (PRV).^[^ ^295^ ^]^ Then they proved that the CdTe QDs can change the framework of viral surface proteins and prevent PRV from entering host cells. Meanwhile, release of Cd^2+^ from CdTe QDs was shown to reduce the quantity of viruses infecting host cells as well. Besides, the size and surface charge of the QDs have extraordinary anti‐PRV performance, and the inhibition effect on the positively charged QDs is higher than that on the negatively charged QDs. In addition, they found that the antivirus ability increases with the QD size.

Moreover, Du et al. have explored the antiviral effects of QDs with less toxicity, such as Ag_2_S nanoclusters (NCs) and carbon dots (CDots).^[^ ^296^, ^297^, ^298^ ^]^ Ag_2_S NCs have been shown to have excellent virus inhibition.^[^ ^298^ ^]^ The porcine reproductive and respiratory syndrome virus (PRRSV) and porcine epidemic diarrhea virus (PEDV) were utilized as RNA and DNA virus models to study the antiviral effect of Ag_2_S NCs with low toxicity. The results showed that Ag_2_S NCs could inhibit the protein expression of PEDV and PRRSV. On the other hand, Ag_2_S NCs significantly induced endogenous IFN production and ISG expression, which is responsible for viral replication. Furthermore, their experiments illustrated that CDots could considerably restrict the proliferation of PRRSV and PRV.^[^ ^296^ ^]^ CDots also significantly induced endogenous interferon production and the expression of interferon‐stimulating genes (ISGs), and these two processes both restrict viral replication.^[^ ^297^ ^]^ Huang et al. demonstrated the benzoxazine monomer derived carbon dots (BZM‐CDots) bind directly to the surface of the virus using non‐enveloped viruses and flaviviruses, and they can restrain the initial stage of interaction between virus and host cell, verifying the virus‐killing ability of functional CDots.^[^ ^299^ ^]^

Therefore, it is believed that QDs have broad prospects as potential antiviral mask material for its antiviral effect. However, its applications for mask production are still needed to be further explored.

### 4.9 Other Improvements on Masks

**Figure 14:**
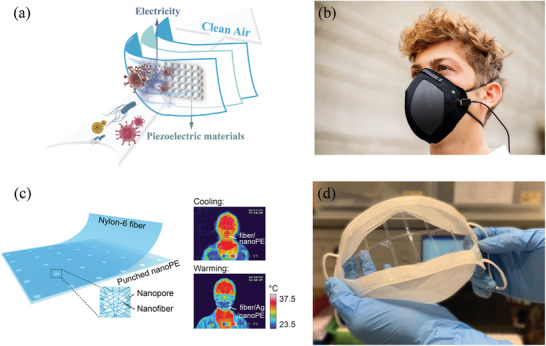
Various applications with different modifications to face masks. a) The proposed concept and design of masks with piezoelectric generator. b) LIG self‐sterilizing mask can be connected with a portable battery via a USB port. b) Reproduced with permission.^[^ ^300^ ^]^ Copyright 2020, Deezen. (c) Masks with thermal management abilities, such as cooling effect and warming effect. Adapted with permission.^[^ ^303^ ^]^ Copyright 2017, American Chemical Society. d) Transparent face mask made of ultrastrong, ultratransparent, cellular, and ultralight films. Adapted under the terms of the Creative Commons Attribution 4.0 International License.^[^ ^305^ ^]^

Apart from the state‐of‐the‐art modifications to masks and respirators discussed above, there have been numerous other attempts and investigations to develop additional functionalities in masks, for instance piezoelectric generator (PEG), self‐sanitizing ability,^[^ ^300^ ^]^ self‐cleaning ability,^[^ ^301^ ^]^ thermal stability management,^[^ ^302^, ^303^, ^304^ ^]^ etc. A piezoelectric generator can also generate electrical power through the use of piezoelectric materials which can generate electricity upon encountering vibrations, and, therefore, it may be possible to utilize a piezoelectric generator in a face mask to produce electrostatic charge which will prolong the lifespan of the mask, much like TENG, as shown in **Figure** **14**a. The self‐sanitizing ability of materials has been developed through various studies which are primarily based on graphene and its derivatives, in which the masks can be heated by solar energy or electricity to kill off viruses, as previously discussed in Section 4.3. Moreover, after the outbreak of COVID‐19 pandemic, Arnusch et al. have attempted to make this technique commercialized by using a type of LIG self‐sterilizing mask, which can be connected to a mobile battery or a home‐based charging system through a USB port, the power of which can be then used to fully sterilize the mask for safe reuse, as shown in Figure 14b.^[^ ^300^ ^]^ In 2017, the idea to integrate thermal management into face masks was initially introduced by Yang and Cui to improve the thermal comfort for mask users.^[^ ^303^ ^]^ They developed a nanofiber‐based face mask using nanoporous polyethylene (fiber/nanoPE) that showed an excellent radiative cooling effect, and the additional inclusion of a further layer of Ag‐coating onto the nanoPE resulted in a warming effect, as shown in Figure 14c. Furthermore, in recent times, an innovative film has been developed that is not only ultratransparent and cellular, but also ultralight and ultrastrong.^[^ ^305^ ^]^ It can achieve a thickness down to 20 nm and has the potential to be fabricated into a transparent face mask that has a good breathability and filtration efficiency, as shown in Figure 14d.

Some, or perhaps even all, of the above modifications may be combined into one mask due to the multifunctional nature of some materials. For example, masks modified with graphene result in a superhydrophobic surface that possesses both antiviral properties and the self‐cleaning ability,^[^ ^216^ ^]^ while masks modified with graphene oxide and TENG possess both antiviral properties and higher filtration efficiencies. Although much research has been done about antimicrobial substances and their corresponding applications, the combination of antimicrobial materials with masks is still in the initial phase of development, especially those involving antimicrobial nanoparticles. In consideration of the increasingly serious COVID‐19 pandemic, more attention should be paid to the development of these masks with antimicrobial properties. Furthermore, as different markets may demand different mask functionalities, there is a growing incentive to both improve pre‐existing technologies and to develop new ones, thereby giving researchers and manufactures new avenues for further exploration.

## 5 Perspective

Due to issues of asymptomatic carriers along with viral residues that remain viable within air aerosols, it is very difficult to limit the aerosol‐transmission of the SARS‐CoV‐2 unless one properly wears a face mask. As the COVID‐19 pandemic becomes a more serious and global problem, face masks and respirators are still expected to be a necessity and thus face high demands. Therefore, it is extremely significant that these demands are met in order to provide adequate protection to people as well as to reduce the further spread of the virus.

However, most fabrics used in cloth masks may not possess the filtration performances needed to protect users as effectively as medical masks. Nevertheless, there are many mature electrostatic charging technologies which have been applied to fabricate commercial masks,^[^ ^62^ ^]^ and hence have the potential to be applied to cloth masks to increase its filtration efficiency. Using a combination of these electrostatic charging technologies and appropriately increasing the number of layers and types of fabrics, particularly cotton, may potentially lead to a >95% filtration efficiency of cloth masks.^[^ ^90^ ^]^ Based on this, cloth masks can be expected to possess more applications. 3D‐printed mask, another substitute, is recyclable, reusable, can prolong mask lifetime, and can potentially address environmental concerns regarding medical waste as only the filter layer is disposable. However, due to the gaps found at the interfaces of different parts, the filtration efficiencies of 3D‐printed masks are usually <90%.^[^ ^132^, ^140^ ^]^ This issue can be solved by either further enhancing 3D printing accuracy to reduce gaps and leakages between different parts altogether or printing the mask directly as a single component instead of as multiple components that require assembly. As a low‐cost and rapid manufacturing technology, 3D printing is regarded as an application with great potential.

Besides direct substitutions for mask materials, masks that possess the ability to kill viruses and retain their filtration efficiencies in the long‐term can be a more effective solution in stopping the spread of the virus. Based on this, many attempts have been made to modify face masks with some antimicrobial materials and other technologies, examples of which include metals and metal oxides, antiviral chemical compounds, graphene‐related materials, MOFs, bio‐based materials, herbal extracts, TENGs, etc. Even so, the integration of antimicrobial substances, especially antiviral substances, into masks or air filters is still at the early stage of its development. Furthermore, there have only been a few studies that have tested the antiviral performance of these emerging materials against COVID‐19 itself. Nevertheless, given the severity of COVID‐19 pandemic, there is a critical need for masks and air filters with antibacterial and antiviral properties. In order for masks to attain this high antibacterial and antiviral performance while also maintaining their biological safety and viability, it is key for the mask material to be able to self‐clean, possess excellent permeability in its structural design, and have a simple and economically viable fabrication method. These key traits will ensure that a mask design can be successfully commercialized while also reducing the risks of secondary health pollution on public health. Technologies such as TENG and PEG, which can not only improve filtration efficiencies of masks but also prolong their lifetime by maintaining their electrostatic charge, are promising for potential application in the future. Nevertheless, it is noteworthy that the use of PEG as a solution is proposed here and has yet to be investigated further.

Integrating masks with these antiviral materials and technologies can not only prevent SARS‐CoV‐2 and other pathogens from spreading, but also prolong the lifetime of masks and thereby reduce waste and other negative environmental effects from the constant disposal of single‐use masks. With the development of different demands across the global market, various masks with other functions in mind have been in development, such as masks that are thermally managed or transparent. We predict that smart multifunctional masks like smart fabrics will play a vital role in our future life.

Moreover, reuse of face masks and respirators is also one of the effective means to alleviate the shortage situation, and meanwhile reduce the medical waste and resultant environmental impact. Recently, researchers and organizations have investigated how to effectively reuse masks.^[^ ^5^, ^120^, ^121^, ^306^, ^307^, ^308^, ^309^, ^310^, ^311^, ^312^, ^313^ ^]^ The key to mask reuse is to inactivate the pathogens that have accumulated on the mask, which involves a decontamination process such that the pathogens are sterilized or removed while still preserving the structural integrity of the mask. This maintains its filtration efficiency, allowing it be safe for wearer to reuse.^[^ ^308^ ^]^ There are various methods that have been developed and are now being used to inactivate microorganisms and disinfect or sterilize equipment in hospitals, laboratories, and other critical institutions.^[^ ^314^, ^315^ ^]^ The United States Centers for Disease Control and Prevention (CDC) advocates several disinfection or sterilization approaches, mainly including chemical solutions (alcohols, peroxides, chlorides, oxidizers, phenolics, aldehydes), radiation of different forms (UV, ionizing radiation, infrared radiation), and temperature treatments (pasteurization, steam, dry‐heat).^[^ ^5^, ^315^ ^]^ As mentioned in Section 1.2, the electrostatic charges in the filter layer play an important role in filtering out and adsorbing fine particles. However, water molecules in the environment may be adsorbed onto the fiber surfaces and result in the loss of electrostatic charges, which then decreases the filtration efficiency of masks.^[^ ^5^, ^78^ ^]^ Therefore, masks should be dried thoroughly after the decontamination process to ensure that high performance is maintained.

However, as of the time of writing, the disposable PPE‐type masks which are currently in use for face masks and respirators, have generated and are generating plenty of waste, and resultant environmental problems have occurred,^[^ ^316^ ^]^ as shown in Figures S1 and S2, Supporting Information. We evaluate the sustainability issues related to usage of disposal of masks using the traditional materials, and our life cycle assessment (LCA) indicates that disposal of 40 000 000 face masks per day significantly contribute to global warming, acidification, and ecotoxicity (detailed analysis is shown in Supplementary Materials). How to deal with this waste has become a serious environmental issue which requires global cooperation and a joint effort.^[^ ^309^, ^317^ ^]^ Based on this, the above studies on reusable masks have positive effects on decreasing the PPE wastes for the time being.

## 6 Conclusions

The rapid outbreak of the COVID‐19 pandemic has brought a terrible disaster to the world. SARS‐CoV‐2, the primary agent of this disease, has a longer incubation period than other viruses that have appeared in the past. Compounded by the existence of asymptomatic carriers of the virus, the problems have dramatically increased due to the difficulty in detecting and monitoring its spread. There are three main transmission pathways for COVID‐19 which are contact, fomites and aerosol transmission. The former two transmission pathways can be mostly avoided by establishing better personal hygiene habits and compulsory quarantine, while the spread of the virus through the latter transmission pathway can be effectively prevented by wearing masks, a protocol that is recommended by the WHO and, indeed, is mandatory in most countries. However, as the global COVID‐19 pandemic is becoming increasingly severe, the supply of masks has fallen far short of the demand in many countries and regions. Based on this situation, this review takes the perspective of recent developments in material technology and analyzes and summarizes various substitutes, antiviral materials and technologies that can enhance the performance of masks and consequently, reveal possible solutions in alleviating the current severe shortage of masks.

As easily available alternatives, cloth masks, and 3D‐printed masks are discussed along with their advantages and disadvantages. Although most of their filtration efficiencies are not as high as 95% required in commercial face masks and respirators, they can help alleviate the current critical shortage and provide some protection against the spread of the virus. More importantly, they have a significant potential to achieve higher filtration efficiencies if some of their aspects are improved. Methods to improve the filtration efficiency of masks, including using nanofibers and introducing other polymer layers into them, are elaborated.

Recent progresses of various materials and technologies that can be used to inactivate viruses and prolong the lifespan of masks are detailed. The advances in material technology have led to the development of antimicrobial coatings and several of these are introduced in this review, namely, metals and metal oxides, antiviral chemical compounds, graphene‐related materials, MOFs, bio‐based materials, herbal extracts and TENGs. When incorporated into masks, these materials and technologies can aid in the prevention of secondary transmission of the virus due to inappropriate mask disposal. However, these technologies are not yet mature enough, and therefore, further studies and innovations must be made to develop multifunctional masks with antiviral and other properties. For instance, the development of thermochromic materials has the potential to allow people to easily recognize individuals with latent symptoms.

Besides these advanced technologies, this review also presents the LCA and comparative results of surgical and N95 masks taking Hong Kong region as the research object to reveal negative environmental impacts of using disposable masks, indicating that it not only consumes much energy, but also has the potential to contribute to global warming, acidification and ecotoxicity. Compared to surgical mask, N95 mask has relatively more adverse environmental effects on all of the five impact categories due to its need for more raw materials and intensive fabrication processes.

Finally, in consideration of the environmental impacts of waste produced from the current widespread use of disposable masks, the use of substitutes or multifunctional modifications can have a positive effect on reducing overall mask waste and protecting our environment. As mentioned before, mask‐wearing is still a key strategy for preventing airborne diseases and hence cannot be easily replaced. Given this, the least we can do is to further improve upon such a ubiquitous technology.

## Conflict of Interest

The authors declare no conflict of interest.

## Supporting information

Supporting Information
