# Supplementary material for: Masks for COVID‐19
Source: Adv Sci (Weinh). 2021 Nov 26;9(3):2102189. doi: 10.1002/advs.202102189 (PMC8787406; doi:10.1002/advs.202102189)
Supplement: Supplementary file 1 — Supporting Information [file ADVS-9-2102189-s001.pdf]

© 2021 Wiley-VCH GmbH

ADVANCED SCIENCE
Open Access

Supporting Information

for *Adv. Sci.*, DOI: 10.1002/advs.202102189

Masks for COVID-19

Wei Deng[#1], Yajun Sun[#1], Xiaoxue Yao[#1], Karpagam Subramanian[#2], Chen Ling[1], Hongbo Wang[1], Shauhrat S. Chopra[2], Ben Bin Xu[3], Jie-Xin Wang[4], Jian-Feng Chen[4], Dan Wang*[4], Honeyfer Amancio[5], Stevin Pramana[6], Ruquan Ye*[7], Steven Wang*[1,2]*

Supplementary Materials

Wei Deng[#1], Yajun Sun[#1], Xiaoxue Yao[#1], Karpagam Subramanian[#2], Chen Ling[1], Hongbo Wang[1], Shaurat S. Chopra[2], Ben Bin Xu*[3], Jie-Xin Wang[4], Jian-Feng Chen[4], Dan Wang*[4], Honeyfer Amancio[5], Stevin Pramana[6], Ruquan Ye*[7], Steven Wang*[1,2]

1. Department of Mechanical Engineering, City University of Hong Kong, Hong Kong, China
2. School of Energy and Environment, City University of Hong Kong, Hong Kong, China
3. Department of Mechanical and Construction Engineering, Northumbria University, Newcastle upon Tyne, United Kingdom
4. State Key Laboratory of Organic Inorganic Composites, Beijing University of Chemical Technology, Beijing, China
5. Department of Chemical Engineering, Cambridge University, Cambridge, United Kingdom
6. School of Engineering, Newcastle University, Newcastle upon Tyne, United Kingdom
7. Department of Chemistry, City University of Hong Kong, Hong Kong, China

Corresponding authors: wangdan@mail.buct.edu.cn; ben.xu@northumbria.ac.uk; ruquanye@cityu.edu.hk; steven.wang@cityu.edu.hk

In addition, during this global pandemic disease, thousands of tons of waste from disposable face masks are produced every day all over the world, which would cause huge damage to our environment without proper disposal [244, 323-327]. Increasing consciousness of the negative environmental impacts of using disposable masks calls for the inclusion of a life cycle assessment (LCA) as part of the decision-making process for mask selection. Here we present a LCA and comparative results of surgical and N95 masks taking Hong Kong region as the research object. The LCA was carried out in four steps: 1) Goal and scope definition, 2) Life cycle inventory, 3) Life cycle impact assessment and 4) Results interpretation.

The primary aim of the LCA is to evaluate the environmental sustainability of the two masks right from the design stage itself. The basis of calculation and comparison, i.e. the functional unit (FU), is taken to be one single-use mask. This is a cradle-to-grave study, as shown in Figure S1, that seeks to determine the global warming potential, energy demand, and other environmental impacts of the production, use, and disposal of two masks. Secondary sources are employed to build the life cycle inventory (LCI) which includes aspects such as the material composition, production processes, and

manufacturing locations. Identified materials and processes are matched with appropriate LCI records in the Ecoinvent v3.7 database. The five basic and important impact categories, namely, (i) the global warming potential (GWP), (ii) energy demand, (iii) acidification potential (AP), (iv) eutrophication potential (EP), and (v) ecotoxicity (ET), are estimated using the Simapro software. Energy demand is calculated using a combination of the single issue impact assessment method, cumulative energy demand (CED), and the ReCiPe (2016) method.

According to the results of LCA, five impact categories of one surgical or N95 mask are shown in Table 3. With regard to CED, the fossil fuel-based energy demand of one surgical mask is 46.29 MJ over its entire life cycle, while the N95 mask requires 225.64 MJ, about five times more. The most considerable energy demand for the N95 mask is its process energy, i.e., the electricity used in the production processes involved in fabricating the mask. The most energy-intensive processes are meltblowing, cup mask forming, and ultrasonic welding, which is used for attaching the nose clips and earloops to the mask. Other processes like spun bonding, nose clip cutting, earloop cutting, ethylene oxide disinfection, assembling, finishing and packaging are less energy-intensive. However, they still contribute to the overall impact on CED and other measures. The majority of the carbon emissions that contribute to the GWP of a N95 mask comes from the electricity needed to produce it, which was assumed to predominantly originate from the burning of fossil fuels. Indeed, since all the production happens in China, the related electricity mix was used for modelling the emissions generated from electricity consumption of producing both types of masks. Because coal is the primary fuel in this electricity mix, which generates a significant amount of carbon emissions compared to other power sources, the resultant environmental impacts for both masks became much higher both in terms of fossil fuel-based energy demand and carbon emissions. Therefore, the results of this LCA may vary for mask production at different geographic locations based on a specific region’s electricity production mix and sources of energy.

Compared to N95 masks, surgical masks were found to have a more favourable environmental profile with relatively fewer adverse environmental effects on all of the five impact categories. The environmental impact category that displayed the largest difference between N95 and surgical masks was that of CED, followed by GWP. Despite the overall lower adverse environmental impact of surgical masks, it continues to produce thousands of tons of waste every single day, more than N95 masks, and resultant environmental impact can be even worse. Identical to N95 masks, the main sources of negative environmental impact of surgical masks come from the electricity used in the production and packaging of these masks. Thus, upgrading to more energy-efficient production and packaging machineries, technologies and systems can further reduce these impacts. Specifically, the CED for surgical mask packaging is 3.98 MJ, with the equivalent N95 mask demand being 1.85 MJ. Nevertheless, the negative environmental impacts that arise from the other production processes of fabricating N95 masks far outweigh this positive impact. For both mask options, the material composition (i.e., non-woven polypropylene, poly bags used in packaging) and transportation do not add significantly to the overall impacts.

Overall, the results shown in Table S1 demonstrate that a single disposable mask not only consumes much more energy, but also has the potential to contribute to global warming, acidification and ecotoxicity; it has been estimated that more than 4 billion disposable masks are used per day worldwide

[328]. This could have an unimaginable negative impact on our environment, which is illustrated as shown in Figure S2. Hereinto, the plastic in disposable masks could take up to hundreds of years to degrade if they finally end up in the sea [329, 330].

In Hong Kong, people mostly prefer single-use surgical masks over other types of face masks [331]. The environmental bureau (ENB) of Hong Kong has estimated that around 4-6 million face masks are being used and disposed off in landfills everyday [332]. On this basis, if 80% of people wear single-use surgical masks, it will account for about 17-25 thousand tons of CO_2-eq of greenhouse gases over its life cycle, and the remaining 20% as N95 will account for about 21-31 thousand tons of CO_2-eq. However, due to the shortage or the financial problem, some people may tend to reuse these masks despite them being originally intended for single-use. Therefore, to validate these estimations, city level data on mask reuse and people's preferences among the two mask options can be included as additional factors in the LCA to better understand its contribution to environmental sustainability.

Many recent studies have shown, as discussed in Section 2-4, that realizing the possibility of reusable mask development through material innovation and technological advances can address the current mask shortage and meanwhile reduce greenhouse gas emissions and negative environmental impacts. Nevertheless, continued efforts are necessary for developing new environmentally-friendly mask materials with additional functionalities, including self-disinfecting ability and biodegradability, as well as to develop new low-energy technologies, processes and systems for the fabrication of these masks.

Table S1. Global warming potential (GWP), energy demand, acidification potential (AP), eutrophication potential (EP) and ecotoxicity (ET) of one surgical mask or N95 mask as estimated using the Simapro software.

			CED (MJ)	GWP (kg CO_2-eq)	AP (kg SO_2-eq)	EP (kg N-eq)	ET (kg 1,4-DCB-Eq)
Surgical mask	Fabrication Process	Non-woven polypropylene	0.166785	0.005306	0.001043	8.28E-07	0.001641
		Polyester	0.056476	0.002656	0.000526	1.30E-06	0.001309
		HDPE (nose clip)	0.003663	0.000107	1.98E-05	9.60E-09	3.16E-05
		Ethylene oxide	1.75E-06	6.16E-08	1.11E-08	5.38E-12	1.77E-08
		Transport	0.034540	0.001980	0.000858	1.19E-06	0.00132
		electricity	42.04460	4.391920	1.240120	0.000624	1.256600
		Packaging	3.985778	0.416201	0.117484	5.99E-05	0.119224
		Landfill	0.000234	1.58E-05	6.32E-06	1.11E-08	3.08E-06
		**Total**	**46.29208**	**4.818186**	**1.360057**	**0.000688**	**1.380129**
	Packaging	electricity	3.979950	0.415740	0.117390	5.91E-05	0.118950
		Corrugated box	0.005515	0.000451	9.21E-05	7.52E-07	0.000271
		Packaging film (LDPE)	0.000313	9.64E-06	1.87E-06	1.20E-09	3.02E-06
		**Total**	**3.985778**	**0.416201**	**0.117484**	**5.99E-05**	**0.119224**
N95 mask	Fabrication Process	Non-woven polypropylene	0.259444	0.008254	0.001623	1.29E-06	0.002553
		Ethylene oxide	4.20E-06	1.48E-07	2.67E-08	1.29E-11	4.26E-08
		Aluminum	0.054521	0.005036	0.001404	7.43E-07	0.002666

		Steel	0.048013	0.004304	0.001287	8.18E-07	0.007021
		Transport	1.439690	0.082530	0.035763	4.95E-05	0.055020
		electricity	221.8567	23.17484	6.543740	0.003294	6.630700
		Earloop (PP)	0.137472	0.004374	0.000860	6.82E-07	0.001353
		Packaging	1.851649	0.192991	0.054406	2.90E-05	0.055551
		Landfill	0.000550	3.78E-05	1.51E-05	2.65E-08	7.38E-06
		**Total**	**225.6480**	**23.47237**	**6.639099**	**0.003377**	**6.754872**
	Packaging	electricity	1.836900	0.191880	0.054180	2.73E-05	0.054900
		Corrugated box	0.012873	0.001053	0.000215	1.76E-06	0.000633
		Packaging film (LDPE)	0.001876	5.79E-05	1.12E-05	7.20E-09	1.81E-05
		**Total**	**1.851649**	**0.192991**	**0.054406**	**2.90E-05**	**0.055551**

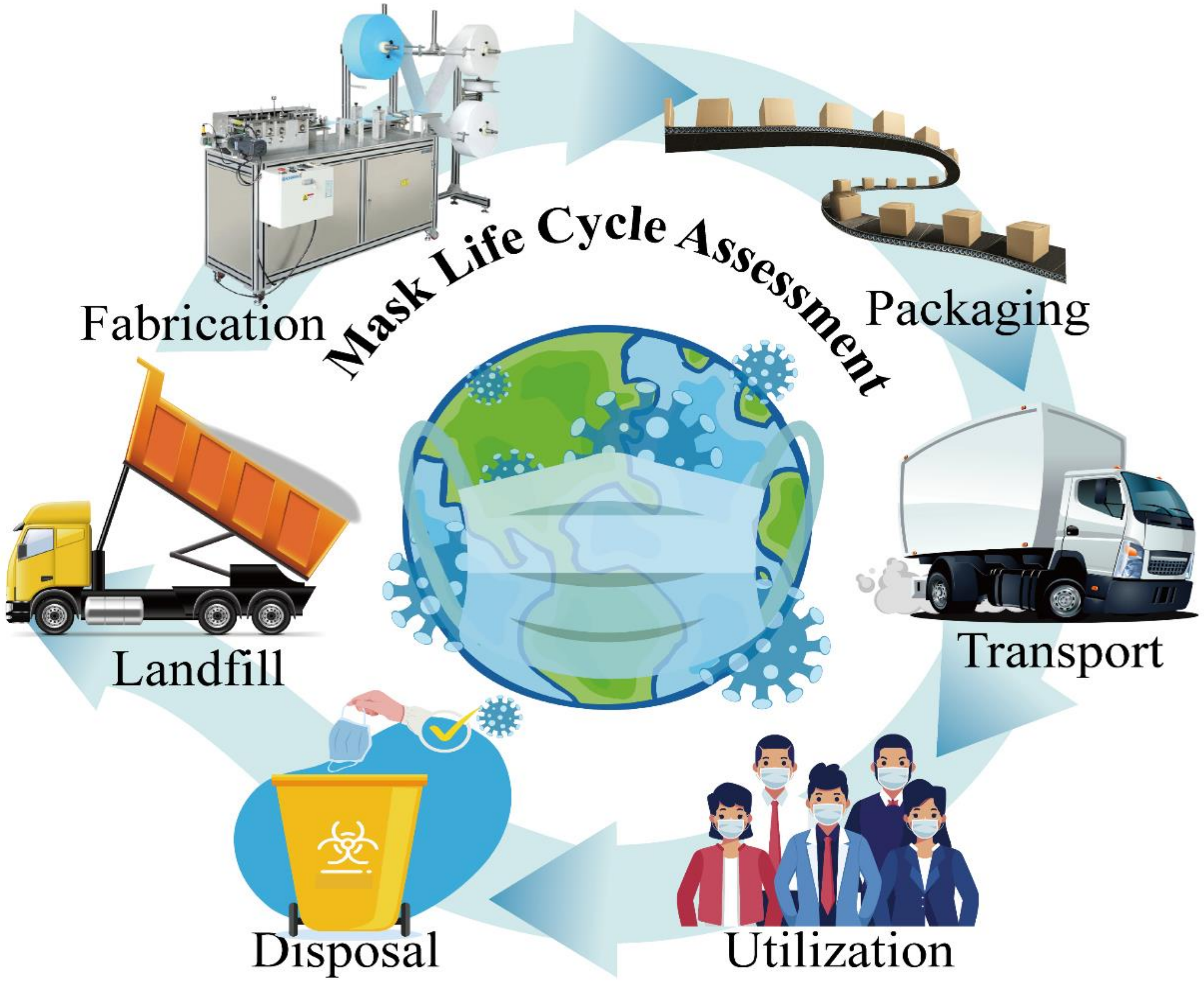

Figure S1. Schematic illustration of cradle-to-grave life cycle of disposable masks.

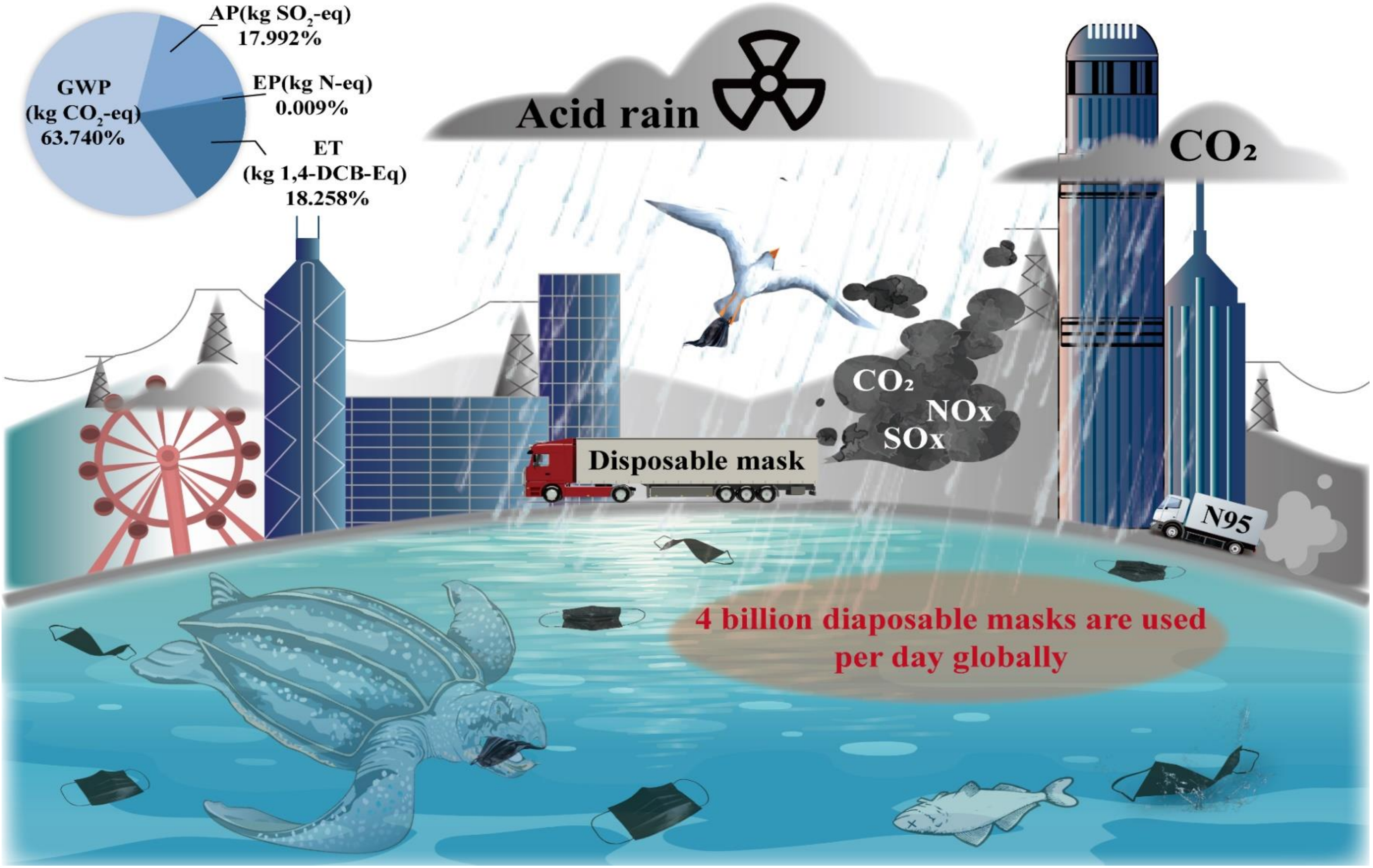

Figure S2. The potential negative environmental impacts of using disposable masks.
